# Oxidative Stress in Health and Disease: Mechanisms and Therapeutic Perspectives

**DOI:** 10.3390/ijms27062681

**Published:** 2026-03-15

**Authors:** Shehwaz Anwar, Hajed Obaid A. Alharbi, Ali Yousif Babiker, Arshad Husain Rahmani

**Affiliations:** 1 Department of Medical Laboratories, Mohan Institute of Nursing and Paramedical Sciences, Bareilly 243302, India; shehwazanwar25@gmail.com; 2 Department of Medical Laboratories, College of Applied Medical Sciences, Qassim University, Buraydah 51452, Saudi Arabia

**Keywords:** oxidative stress, disease, health, cancer, chronic disease, pathogenesis

## Abstract

Reactive oxygen species (ROS) are inevitable byproducts of aerobic metabolism that exert a dual role in biological systems. At physiological levels, tightly regulated ROS levels function as essential signaling molecules regulating cellular communication, immune defense, metabolic adaptation, and maintenance of tissue homeostasis. However, excessive or deregulated ROS production disrupts redox balance and contributes to oxidative stress, a key factor in the onset and progression of numerous pathogenesis. This review provides an updated and integrated overview of ROS biology, summarizing their major types, cellular and molecular sources, and physiological functions, highlighting their significance in physiological redox signaling and oxidative stress-mediated disease mechanisms. Key molecular pathways involved in ROS-induced cell damage, redox imbalance, and signaling dysregulation are discussed. In addition, contemporary and emerging approaches for the detection and quantification of ROS and oxidative stress in clinical and preclinical samples—such as biochemical assays, fluorescent probes, biosensors, and advanced imaging techniques—are critically evaluated. The contribution of oxidative stress to the pathophysiology of major disorders, including cancer, diabetes, cardiovascular diseases, neurodegenerative conditions, and inflammatory disorders, is also examined. Finally, this review highlights future perspectives in precision redox medicine, emphasizing the potential of targeted antioxidant-based diagnostic and therapeutic strategies supported by advances in ROS detection technologies and a deeper understanding of redox-regulated biological processes.

## 1. Introduction

Electron transfer (redox) reactions that deviate from thermodynamic equilibrium mediate a wide range of physiological processes in living organisms. These redox reactions are involved in cell bioenergetics, oxidative phosphorylation, DNA damage, enzymatic catalysis, and drug metabolism. Since electrons are continuously transferred between molecules during these reactions, free radicals are continuously produced as an intermediate species [1]. Among these, reactive oxygen species (ROS) are highly reactive oxygen-containing chemical entities constantly produced by the body during cellular metabolism [2,3,4]. ROS comprise a diverse group of oxidant molecules with markedly different chemical properties and biological functions, ranging from regulatory signaling roles to the induction of cellular damage and death [5].

Under physiological conditions, ROS and reactive species, such as reactive nitrogen species (RNS) and reactive sulfur species, are maintained at low levels in tissues by natural antioxidant defenses. However, when their production exceeds their clearance, an imbalance, i.e., oxidative stress, occurs. This condition disrupts redox signaling, leads to oxidative modification of biomolecules, and impairs cellular function, contributing to numerous diseases. ROS are continuously produced during normal metabolic activities at low levels. Acute or chronic cellular stress, as well as external factors, such as ozone, air pollution, cigarette smoke, pathogens, medicines, and radiation (UV and X-rays), can enhance the production of ROS [6]. Molecular oxygen contains two unpaired electrons in its outer orbit, making it a biradical capable of generating several ROS. Among these, superoxide (O_2_^−•^) and hydroxyl radical (OH^•^) are especially important. OH^•^ is extremely reactive yet short-lived, persisting only for a billionth of a second. Other ROS, like alkoxyl (RO^•^) and peroxyl (ROO^•^) radicals, though less reactive, play key roles in lipid peroxidation. Nitric oxide (NO^•^), a relatively stable signaling molecule, can rapidly react with O_2_^−•^ to form peroxynitrite (ONOO^−^), which subsequently decomposes into OH^•^ and nitrogen dioxide (^•^NO_2_) [7].

ROS are produced primarily from the incomplete reduction in molecular oxygen during normal metabolism and may exist either as free radicals with unpaired electrons or as non-radical species formed through radical–radical interactions. ROS can damage macromolecules such as DNA, proteins, and lipids due to their high reactivity [8]. Various enzymatic reactions continuously produce ROS and serve essential functions as second messengers in redox signaling. Their interactions with redox-sensitive amino acid residues, such as cysteine, modulate the activities of transcription factors (e.g., AP-1, NF-κB, and HIF-1) and various enzymes (e.g., protein tyrosine phosphatases). These affected cellular processes include proliferation, differentiation, growth arrest or senescence, and cell death [9,10]. The specificity of ROS signaling depends on the site of generation, spatial distribution, pulse concentration, and temporal duration [10,11,12].

Various cell types have different ROS-generating enzyme expression, localization, and regulation mechanisms, as ROS production differs among them. DUOX1/DUOX2 is expressed in non-phagocytic cells and tends to produce H_2_O_2_. NOX2 enzyme is highly expressed in phagocytic cells, such as neutrophils and macrophages, to generate O_2_^−•^ during host defense. The NADPH oxidase 2 (NOX2) is highly expressed in phagocytic cells, such as neutrophils and macrophages, and the NOX family consists of seven isoforms with different cellular distributions and ROS outputs. The subcellular location and activity of these isoforms also affect the kind and quantity of ROS generation by various cell types [13,14].

The balance between ROS formation and antioxidant defenses determines the transition from physiological to pathological ROS and is not a fixed numerical threshold. At low-to-moderate levels, ROS function as signaling molecules, which are implicated in regulation of cell proliferation, differentiation, immune responses, and metabolic adaptation. This balance is governed by crucial regulatory processes, including the activation of redox-sensitive transition factors like Nrf2, NADPH oxidase activity, mitochondrial control of ROS generation, and the larger cellular antioxidant network. Together, these mechanisms preserve redox homeostasis and impact downstream gene expression and cellular outcomes [15].

**Figure 1:**
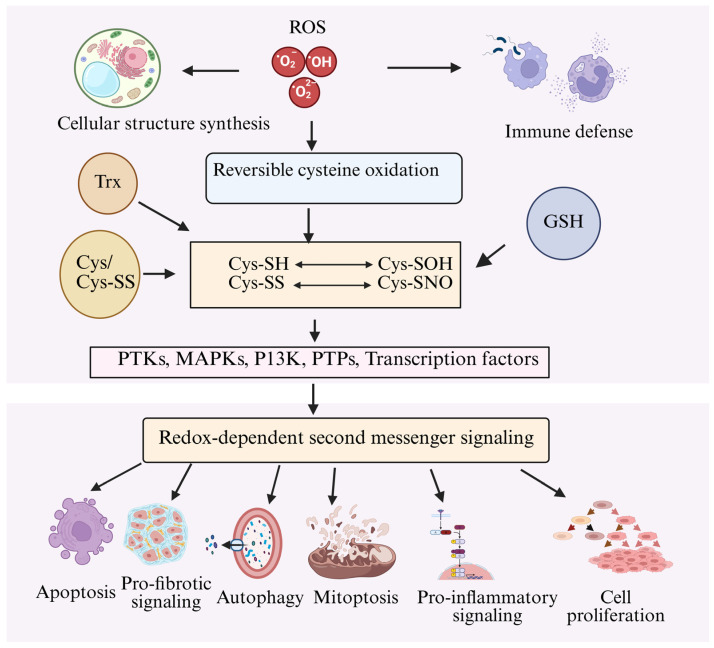
Physiological ROS signaling via reversible cysteine sulfur switches. Physiological ROS act as second messengers by inducing reversible cysteine oxidation. These redox switches, regulated by Trx, GSH, and the Cys/CySS system, modulate kinases, phosphatases, and transcription factors to control immune defense, cellular structure synthesis, apoptosis, autophagy, mitoptosis, pro-inflammatory/pro-fibrotic signaling, and cell proliferation. Created in BioRender. Rahmani, AH. (2026) https://app.biorender.com/illustrations/canvas-beta/698a78d8237a9787ff943cec, accessed on 5 March 2026.

It appears that ROS were selected by nature as signaling mechanisms early in evolution so that they could respond to alterations in the oxidative environment and environmental nutrients. In reality, there are well-established processes in prokaryotes by which ROS directly activate transcription factors to aid in stress adaptation. Thus, ROS’s physiological and pathological functions are both included in redox biology. The main question in redox biology is still whether to utilize antioxidant therapy to avoid ROS-linked pathologies or enhance ROS signaling for adaptive responses [16,17]. ROS are well documented as signaling agents, and this role was postulated over 50 years ago (Proctor PH). Beyond classical targets, ROS act through orthogonal mechanisms. They can modulate cellular processes without directly affecting canonical signaling pathways. A framework for combining ROS signals with cell cycle, death, and metabolic regulation is provided by cysteine-based redox-sensing in proteins. Selective thiol oxidation, compartment-specific redox states, and localized ROS production allow for both global and precise regulation of biological processes. Knowing these processes reveals ROS to be crucial, context-dependent regulators of metabolism and signaling rather than just harmful substances [18,19] (Figure 1).

**Figure 2:**
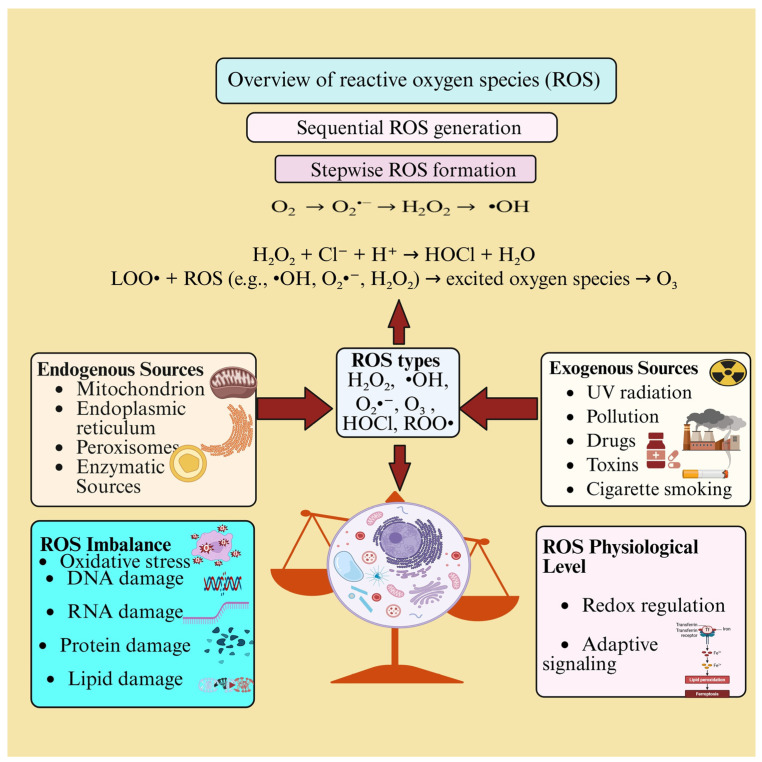
Cellular sources and dual roles of reactive oxygen species (ROS). This figure provides an overview of where ROS come from and how they function inside the cell. It shows both endogenous sources, such as mitochondria, endoplasmic reticulum, peroxisomes, and enzyme systems, and exogenous sources, including UV radiation, pollution, drugs, toxins, and cigarette smoke. The diagram also presents the main types of ROS produced (O_2_^•−^, ^•^OH, H_2_O_2_, ROO^•^, HOCl, and O_3_) and their stepwise formation. Importantly, it highlights the dual nature of ROS: at controlled physiological levels, they support redox signaling and cellular adaptation, whereas excessive ROS disrupt cellular balance and contribute to oxidative damage to DNA, RNA, proteins, and lipids. Created in BioRender. Rahmani, AH. (2026) https://app.biorender.com/illustrations/canvas-beta/695501a4e45259ec219bbfe3, accessed on 22 February 2026.

Multiple intracellular sources, including mitochondria, endoplasmic reticulum, and peroxysomes, as well as specific enzyme clusters, such as NADPH oxidases (NOX), contribute to ROS production [20]. Among these, mitochondrial complexes I, II, and III of the ETC are the major contributors to cellular ROS production [21]. Animal studies from *Caenorhabditis elegans* to mammals demonstrate that DNA damage-response systems coordinate adaptive mechanisms to preserve cellular integrity during aging. However, prolonged oxidative stress progressively overwhelms these defenses [22]. Figure 2 provides an overview of reactive oxygen species (ROS), illustrating their endogenous and exogenous sources, sequential generation, and major ROS types. It highlights the dual role of ROS, where balanced levels regulate redox signaling and adaptive responses, while excess ROS disrupt cellular homeostasis, leading to oxidative stress and damage to DNA, RNA, proteins, and lipids.

Oxidative stress plays a pivotal role in the onset and progression of diverse human diseases, including various cancers, diabetes, cardiovascular diseases, renal dysfunction, and neurodegenerative diseases. In cancer biology, ROS-mediated activation of transcription factors such as MAPKs, AP-1, and NF-kappaB alters gene expression patterns that govern cellular proliferation, apoptosis, and inflammation [23]. For example, oxidant-induced AP-1 activation enhances the expression of cyclin D1 and cyclin-dependent kinases (cdks) and drives cell cycle progression [24], while ROS-mediated NF-κB activation modulates cytokine responses and survival pathways [25]. Accumulation of oxidative DNA damage, base modifications, mispairing, sequence rearrangements, and impaired repair mechanisms leads to neoplastic transformation [26].

Given their dual role in sustaining normal physiology and disease development, ROS have emerged as critical therapeutic agents. Approaches aimed at modulating oxidative stress include natural and synthetic antioxidants, enhancement of endogenous enzymatic defenses, redox signaling modulators, and lifestyle interventions. A detailed understanding of ROS production, cellular redox regulation, and oxidative damage is therefore essential for developing effective prevention and treatment approaches.

Recent advances in redox biology emphasize the ROS function as tightly regulated signaling molecules with spatial and temporal specificity, rather than toxic byproducts of metabolism. However, there are key challenges in distinguishing redox physiological signaling roles from pathological oxidative damage, improving the accuracy of ROS detection, and translating mechanistic insights into effective clinical interventions [27]. Therefore, this review aims to provide a concise, integrated overview of ROS biology by linking their sources, regulatory mechanisms, roles in pathologies, and current approaches for their measurement and therapeutic modulations.

This narrative review integrates recent, highly referenced, and seminal peer-reviewed publications that are pertinent to the issue in order to summarize current knowledge on ROS biology, toxicity mechanisms, and disease connections. Instead of using rigorous systematic inclusion and exclusion criteria, the literature was chosen based on conceptual relevance, mechanistic insight, and translational significance. Important research that has influenced our knowledge of oxidative stress-mediated molecular pathways, redox signaling, and its relevance to human disorders were given special attention.

This review provides an integrated perspective by connecting fundamental ROS biology with disease mechanisms, detection technologies, and therapeutic implications within a single framework. While oxidative stress has been extensively studied, the existing literature often focuses on isolated aspects of redox biology. By consolidating dispersed knowledge and highlighting current challenges in ROS measurement, biomarker interpretation, and targeted antioxidant strategies, this work offers a cross-disciplinary synthesis relevant to both basic and clinical research. This integrated approach helps clarify the translational significance of redox regulation and supports future development of improved diagnostic and therapeutic strategies.

## 2. Methodology

A comprehensive literature search was conducted to collect relevant scientific evidence regarding reactive oxygen species (ROS), oxidative stress, and their roles in physiological and pathological processes. Electronic databases and search engines, including Google, Google Scholar, PubMed, and Scopus, were systematically searched. The search covered publications from November 1991 to December 2025, ensuring the inclusion of both foundational discoveries in free radical biology and the most recent advances in redox research. The following keywords and combinations were used: “Free radicals,” “Reactive oxygen species,” “Sources of ROS,” “ROS signaling,” “Redox signaling,” “Oxidative stress,” “Advanced imaging techniques,” “Oxidative stress biomarkers,” “Cancer and oxidative stress,” “Diabetes and ROS,” “cardiovascular diseases and ROS,” “Neurodegeneration and oxidative stress,” and “Inflammation and ROS.” The initial search yielded approximately 400 references. After removing duplicate and overlapping records, 316 articles were included in the final analysis.

**Inclusion Criteria**

Studies were included if they met the following criteria: Peer-reviewed original research articles, review articles, clinical and preclinical studies, and articles published in English.

**Exclusion Criteria**

The following were excluded: Case reports, editorials, letters to the editor, theses and dissertations, conference abstracts, and non-English publications.

## 3. Free Radicals and ROS

A free radical is a kind of atom, cluster of atoms, or molecule that has an unpaired electron in one of its atomic orbitals and is capable of existing on its own. They are highly unstable and are extremely reactive. Free radicals readily interact with other molecules due to their unpaired electrons. A molecule may become a free radical by losing one of its electrons when it comes into contact with a free radical. This triggers a series of events that causes extensive cell damage. This damage can interfere with regular cellular processes and lead to several diseases [4]. They act as oxidants or reductants by accepting or donating unpaired electrons. While excessive free radicals are harmful, they serve critical physiological roles at low-to-moderate concentrations, such as acting as signaling molecules [28]. Additionally, they can help prevent smooth muscle cell proliferation, regulate systemic circulation, and assist the host defense system in fighting pathogens. In particular, homeostatic regulation is necessary for aerobic cells experiencing the oxygen paradox to maintain free radical levels in metabolism at healthy levels [29]. Table 1 summarizes the classification of major reactive oxygen species (ROS), summarizing their chemical nature, primary sources, and key physiological and toxicological roles in biological systems.

ROS can be classified as radical ROS and non-radical ROS. Examples of radicals include NO^•^, NO_2_^•^, O_2_^−•^, hydroxyl (OH^•^), alkoxyradical (RO^•^), and ROO^•^. One unpaired electron in these radicals tends to donate or acquire another electron in order to achieve stability, which accounts for their high reactivity. Hydrogen peroxide (H_2_O_2_), hypochlorous acid (HOCl), hypobromous acid (HOBr), O_3_, ^1^O_2_, nitrous acid (HNO_2_), nitrosyl cation (NO^+^), nitroxyl anion (NO^−^), dinitrogen trioxide (N_2_O_3_), dinitrogen tetraoxide (N_2_O_4_), nitronium (nitryl) cation (NO_2_^+^), organic peroxides (ROOH), aldehydes (RCHO), and ONOO^−^ are examples of non-radical species [41]. Although they are not free radicals, these non-radical species can readily cause free radical reactions in living things [42]. ROS play a dual role depending on their concentration, cellular compartment, exposure duration, and the surrounding environment. While high levels cause oxidative damage, moderate levels act as secondary messengers in signal transduction, immune defense, and cellular homeostasis [15].

### 3.1. Superoxide Radical

O_2_^•−^ is a primary ROS generated during mitochondrial oxidative phosphorylation when electrons leak from the electron transport chain (ETC) and reduce molecular oxygen. Two oxygen atoms with 17 electrons and a negative electrical charge form O_2_^•−^, the reduced form of molecular oxygen O_2_ [43]. Although mitochondria are the major source, O_2_^•−^ is also generated by several enzymatic systems, including NADPH oxidases (NOX), xanthine oxidase (XO), and cytochrome P450 (CYP)/cytochrome P450 reductase (POR), as well as through non-enzymatic reactions [44].

In biological systems, O_2_^•−^ plays a dual role as a signaling molecule and as a precursor to more reactive oxidants. Its intracellular levels are tightly regulated by superoxide dismutase (SOD) and superoxide reductase (SOR), which convert it into H_2_O_2_ and thereby limit its accumulation. Due to its rapid conversion into secondary ROS, defining its direct contribution to specific pathological processes is often challenging. O_2_^•−^ contributes to cellular toxicity primarily through damage to iron–sulfur (Fe–S) cluster-containing proteins such as aconitase, succinate dehydrogenase, and NADH–ubiquinone oxidoreductase. The release of free iron from these proteins promotes the formation of highly reactive species, including hydroxyl radicals and peroxynitrite, which amplify oxidative damage [43].

### 3.2. Hydroxyl Radical

The hydroxyl radical (^•^OH) is a significant ROS in biological systems and causes damage to important biomolecules, including DNA, proteins, and membranes [45]. Rarely, the immune system can produce hydroxyl radicals as a byproduct. Microglia and macrophages may also produce this radical when they encounter pathogens. Hydroxyl radical has a short life span of 10^−8^ to 10^−9^ s. In biological systems, hydroxyl radicals can be produced by redox-metal-catalyzed breakdown of H_2_O_2_ or, more likely, by homolytic cleavage of water under high-energy irradiation [4]. In vivo, hydroxyl radicals are produced by several processes, including the homolysis of H_2_O_2_, the fission of H_2_O upon exposure to ionizing radiation, and the reaction of specific transition metal ions with H_2_O_2_, particularly ferrous ion (Fe^2+^) and cuprous ion (Cu^+^) (Fenton reaction) [46].

The specific pattern of oxidative DNA damage by ^•^OH in vivo and in isolated cells exposed to oxidative stress has already been demonstrated. ^•^OH plays a significant role in oxidative macromolecule damage, in vivo, when combined with the capacity to trap ^•^OH in living systems using particular techniques [4]. This is partially caused by the production of 8-oxodG, which can also be produced by the attack of ^1^O_2_ and CO_3_^•−^ on DNA, as well as numerous other mutagenic and/or cytotoxic lesions that are created by the attack of ^•^OH on purines, pyrimidines, and 2′-deoxyribose [46].

### 3.3. Hydrogen Peroxide

With an oxygen–oxygen bond, H_2_O_2_ is a relatively stable peroxide molecule that breaks down slowly. The primary source of H_2_O_2_ is the O_2_^•−^ dismutation reaction, which SOD catalyzes. However, it can also be produced by the two-electron reduction of oxygen in reactions catalyzed by oxidases such as glucose oxidase and xanthine oxidase. Because H_2_O_2_ is neutral, it can diffuse through biological membranes and can accumulate within cells due to the slow kinetics of its reaction with many biomolecules [38]. Under conditions of disrupted metal homeostasis, free transition metal ions catalyze the cleavage of H_2_O_2_ to produce highly reactive ^·^OH and OH^−^ through the Fenton reaction [45]. Beyond its role as a precursor of more reactive oxidants, it also functions as an important signaling molecule involved in growth factor-mediated signal transduction, regulation of thiol redox homeostasis, and mitochondrial function [1].

### 3.4. Peroxyl (ROO^•^) and Alkoxyl (RO^•^) Radicals

ROO^•^ and RO^•^ are potent oxidizing agents in biological systems due to their strong electron-accepting tendency and high redox potential (~1000–1600 mV). In fact, the simplest peroxyradical HOO^•^ is the protonated form (conjugate acid, pKa~4.8) of O_2_^•−^ and is commonly referred to as either hydroperoxyl radical or perhydroxyl radical [47]. HOO^•^ plays a key role in initiating lipid peroxidation by abstracting bis-allylic hydrogen atoms from polyunsaturated fatty acids, triggering chain reactions through both lipid hydroperoxide (LOOH)-dependent and independent pathways [48]. These radical-mediated reactions contribute substantially to transition metal-induced lipid peroxidation in biological systems. In addition to lipids, ROO^•^ and related carbon-centered radicals can directly damage biomolecules such as DNA and protein thiol groups (e.g., albumin-SH) and may inactivate enzymes such as lysozyme [49]. Because of their role in propagating oxidative chain reactions, these radicals are frequently used in experimental systems to evaluate antioxidant capacity. Assays based on azo-initiator-induced lipid peroxidation or protein oxidation, such as the TRAP assay, measure the ability of antioxidants to inhibit radical-mediated damage [50].

### 3.5. Hypoxyl Radical

In activated neutrophils, a substantial amount of H_2_O_2_ is converted into hypoxyl radical (HOCl) by the enzyme myeloperoxidase. During infection, neutrophils are rapidly recruited to the affected site, where HOCl is generated as a component of the innate immune response to eliminate invading microorganisms [51,52]. Owing to its low molecular weight and electroneutrality, HOCl readily diffuses across cell walls and membranes and reacts with a wide range of biological molecules, including DNA, RNA, thiols, heme proteins, amino groups, carbohydrates, and lipids. Despite its strong bactericidal effects and its crucial role in the human immune system, excessive production of HOCl also contributes to tissue injury and has been implicated in the pathogenesis of conditions such as atherosclerosis, chronic inflammation, and certain cancers. DNA damage induced by HOCl and related oxidants, including N-chloramines, may partly explain the association between chronic inflammation and tumor development [38]. Hypochlorous acid solutions are also widely recognized for their potent antimicrobial properties and are considered more biocompatible and less corrosive than many chlorine-based disinfectants, with a lower tendency to promote microbial resistance [53]. The importance of HOCl-mediated microbial defense is highlighted in chronic granulomatous disease (CGD), where impaired NADPH oxidase activity reduces ROS production and results in increased susceptibility to recurrent infections [54].

### 3.6. Ozone

Ozone (O_3_) is a highly reactive, oxidative gas linked to poor health outcomes, such as morbidity and mortality. Under normal circumstances, O_3_ is a volatile molecule [55]. Oxidative stress induced by prolonged O_3_ exposure has detrimental effects, including increased ROS levels and a weakened antioxidant defense system [56]. O_3_ has oxidizing qualities and is highly reactive. The body’s redox balance is changed when O_3_ is inhaled. Reactive oxygen species, including O_2_^•−^, H_2_O_2_, ^•^OH, ^•^NO, etc., are produced by O_3_. Changes in cell signaling induced by increased levels of these species result in a loss of control over the inflammatory response and a persistent state of oxidative stress. Since redox signals play a significant role in cell signaling, biological systems must preserve the oxidation–reduction balance. Oxidative stress results from an organism’s loss of oxidation–reduction balance brought on by an excess of oxidants [57]. Because O_3_ is a strong and extremely reactive oxidant, breathing it in causes oxidative stress and inflammatory reactions in the lower respiratory tract [58]. Chronic O_3_ exposure causes a loss of redox balance, which results in the oxidation of proteins, lipids, and DNA, as well as energy failure from a lack of adenosine triphosphate (ATP), epigenetic changes, and ultimately metabolic changes that cause cell death [59].

## 4. Sources of ROS

Both enzymatic and non-enzymatic processes continuously produce ROS. ROS are typically produced physiologically as a consequence of regular cellular metabolism in the mitochondrial respiratory chain’s oxidative reaction pathways. Furthermore, several intracellular and extracellular processes that control homeostasis in the body, including cell division, differentiation, and death, produce ROS. The primary source of oxidative stress is an imbalance in the activity of endogenous pro-oxidant enzymes, including catalase (CAT), glutathione peroxidase (GPx), NADPH oxidase, xanthine oxidase, and mitochondrial SOD [60]. The principal endogenous and exogenous sources of ROS, along with their primary sites of generation and representative reactive species, are summarized in Table 2.

### 4.1. Exogenous Sources of ROS

Several environmental and lifestyle-related factors contribute significantly to the production of ROS and RNS. Cigarette smoke directly contains numerous free radicals and also activates immune cells in the lungs, amplifying oxidative damage [76]. In the liver, alcohol metabolism increases NADPH production, which enhances mitochondrial O_2_^−•^ and H_2_O_2_ formation [77]. Ozone exposure induces lipid peroxidation and inflammatory cell infiltration in the airways, leading to elevated oxidative stress even after short-term exposure [76]. In the presence of oxygen, ionizing radiation generates reactive species such as H_2_O_2_ and ROO^•^, which further drive oxidative stress through Fenton-type reactions with redox-active metals [77]. Xenobiotics such as heavy metals, environmental pollutants, and per- and polyfluoroalkyl substances (PFAS) promote ROS production through Fenton-type reactions, mitochondrial dysfunction, and inhibition of antioxidant enzymes [76,78]. Common drugs such as chemotherapeutic agents and immunosuppressants further elevate ROS levels either as adverse effects or as part of their therapeutic mechanism, particularly in cancer treatment. By increasing free radical production, some immunosuppressive and chemotherapeutic medications can cause oxidative stress. While doxorubicin and cisplatin increase intracellular ROS through mitochondrial redox cycling, metal-mediated processes, and direct radical production, agents such as cyclosporine, tacrolimus, and gentamicin promote lipid peroxidation. By interfering with ROS-regulating systems, pro-oxidant anticancer medications exploit cancer cells’ increased susceptibility to oxidative stress, ultimately resulting in selective cytotoxicity [77]. Collectively, these exogenous factors disrupt redox homeostasis and drive oxidative stress-mediated cellular injury.

### 4.2. Intracellular Sources of ROS

**Figure 3:**
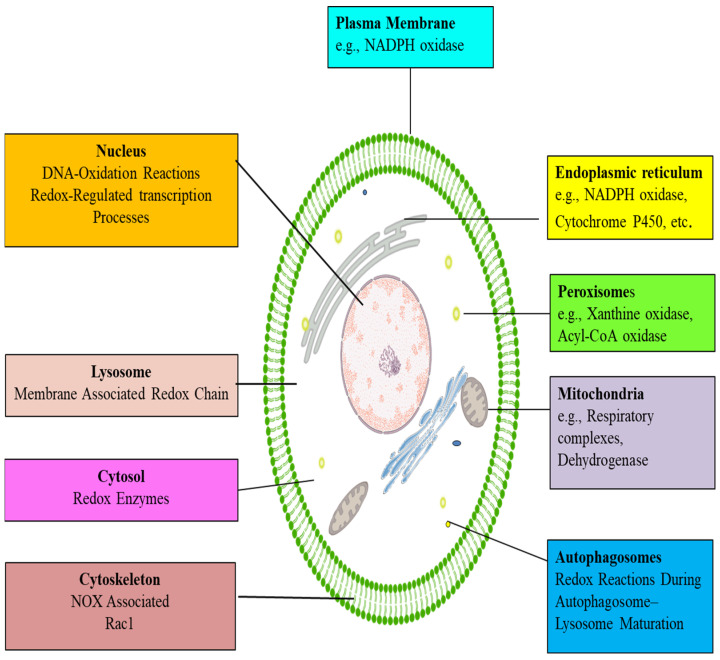
Major intracellular sources of reactive oxygen species (ROS). The Figure summarizes major intracellular ROS generation sites, highlighting how organelles and redox enzymes—including mitochondrial respiratory complexes, NADPH oxidase, cytochrome P450, and xanthine oxidase—produce O_2_^•−^ and H_2_O_2_ through specific enzymatic reactions. The Figure was created by using BioIcons (https://bioicons.com) and Servier Medical Art (https://smart.servier.com).

ROS can originate from a variety of intracellular sources, including the mitochondria and NADPH oxidases (NOX), which are the primary intracellular ROS sources. The endoplasmic reticulum, cytochrome p450, xanthine oxidase (XO), uncoupled NO synthase, peroxidases, and cyclooxygenases are further producers of ROS [37] (Figure 3).

### 4.3. Mitochondrial Dysfunction

O_2_^•−^ radical is the main byproduct of ROS production in mitochondria. The mitochondria contain the oxidation pathways for both fatty acids and carbohydrates. Complex I, complex II, complex III, and complex IV are the four enzyme complexes that aid in the electron transport mechanism that is necessary for the synthesis of ATP, or energy, in mitochondria. The two central locations in the electron transport chain where O_2_^•−^ are produced are complex I (NADH dehydrogenase) and complex III (cytochrome c reductase or ubiquinone). The majority of complex III’s ROS generation can be observed with the antimycin inhibitor A. By moving electrons from NADH dehydrogenase to coenzyme Q, reduced ubiquinone is produced. The mitochondrial enzymes MAO, cytochrome b5 reductase, NADPH oxidase 4, and dihydroorotate dehydrogenase may contribute to ROS production. Other extremely reactive radicals, including carbonate radicals, nitrogen species, and ^1^O_2_, can be produced as a result of ROS. The respiratory process is the primary cause of ROS generation in mitochondria [79].

Under stress, mitochondria produce more ROS, which are linked to inflammatory cytokines. TNF-α, for instance, might reduce complex I’s normal activity, leading to mitochondrial dysfunction and cell death. Furthermore, mitochondrial dysfunction has been shown to enhance cellular susceptibility to a range of pro-inflammatory cytokines and active inflammatory pathways, ultimately leading to increased ROS generation and cellular damage. Additionally, proapoptotic molecules such as cytochrome C and secretory-associated senescence phenotype (SASP), a crucial organelle linked to cell aging and age-related diseases, are released by damaged mitochondria [37].

### 4.4. NADPH Oxidases

One significant producer of ROS, particularly in the cardiovascular system, is NADPH oxidases (NOX). Cardiovascular pathology is facilitated by NOX-mediated excessive ROS generation. Furthermore, many age-related illnesses are linked to NOX overexpression or high activity. Activation, location, and kind of ROS produced by the seven isoforms of NOX that have been found are as follows: NOX1, NOX2, NOX3, NOX4, NOX5, Duox1, and Duox2 [80]. The seven NOX enzymes found in humans have different regulation mechanisms but a very similar catalytic core. The field has advanced with the recent structural elucidation of the NOX catalytic domains. Electrons are transferred to the two sides of the membrane via a linear array of redox cofactors made up of NADPH, FAD, and two hemes. There is no covalent intermediate with the heme iron in the peculiar outer sphere mechanism that reduces oxygen. The functions of NOXs in cell signaling, innate immune response, and cell proliferation—including neoplastic transformation—have been broadened by several recent investigations [81].

All of these NOX family isoforms (NOX1–3, NOX4, and NOX5) are transmembrane proteins that work by moving electrons across biological membranes to lower the amount of oxygen in H_2_O_2_ or O_2_^•−^. When NADPH first binds to the dehydrogenase domain, electrons are successively transferred from the NADPH substrate to the FAD cofactor and, finally, to the two heme groups in the transmembrane domain, generating ROS. Oxygen is the last electron acceptor on the opposite side of the membrane, where it is reduced to produce H_2_O_2_ or O_2_^•−^. Numerous substances, including NADPH oxidase, toll-like receptor agonists, leptin, cytokines (PDGF, TGF-β, and Tnf-α), and hormones, including angiotensin II (Ang II), aldosterone, and endothelin-1 (ET-1), can increase the generation of ROS [37].

Unlike other sources, NOX produces ROS as a byproduct of a primary reaction rather than as a byproduct of a highly controlled, complicated enzymatic activity. NOX members’ production of ROS is controlled by their interactions with a range of membrane-associated and cytoplasmic proteins, as well as by chemical cues such as Ca^2+^ or phorbol 12-myristate 13-acetate. For isoforms 1–3, phosphorylation events initiate the creation of an active NOX enzymatic complex [82].

### 4.5. Xanthine Oxidase and ROS Production

Xanthine oxidoreductase (XOR) belongs to a highly conserved family of molybdo-flavoenzymes that are thought to have shared an ancient progenitor and are found in a wide range of prokaryotic and eukaryotic organisms. The oxidase form of XOR is called xanthine oxidase (XO), whereas the dehydrogenase form is called xanthine dehydrogenase (XDH). The same gene produces both the oxidase and dehydrogenase versions. At the FAD reaction site, XDH produces NADH and has a predilection for NAD^+^ reduction. Conversely, XO does not react with NAD^+^ and uses only dioxygen as a substrate, producing ROS, including H_2_O_2_ and O_2_. ROS, including O_2_^•−^, and H_2_O_2_ are produced while XOR continues to catalyze the oxidation of hypoxanthine and xanthine to uric acid. The reversible conversion of XDH to XO, or vice versa, is of significant interest in pathological settings because it has been linked to diseases that cause tissue damage from oxygen radicals, such as post-ischemic reperfusion injury [83].

The 300 kDa homodimer of the mammalian XOR protein is made up of three domains in each subunit, each of which has unique properties and activities. The NADH oxidase activity occurs in the FAD domain, whereas the substrate pocket for XDH, XO, and nitrite reductase activity of XOR is located in the largest domain. XOR functions as a detoxifying and drug-metabolizing enzyme because of its low substrate specificity and extremely flexible activity, which enable it to oxidize and reduce a variety of endogenous and exogenous products [84].

By directly delivering electrons to molecular oxygen, XO produces H_2_O_2_, O_2_^•−^, and ROS through one-electron and two-electron reductions, respectively. Through the Haber–Weiss and Fenton reactions, this produces the hydroxyl radical (^•^HO) when iron is present. O_2_ tension, pH, and purine content all affect the proportion of divalent versus univalent electron transfer to O_2_, as well as the relative amounts of mathematical equation and H_2_O_2_ produced by XO. Under hypoxic conditions, XDH can also create these ROS by oxidizing NADH at the FAD site. Low O_2_ tension and hypoxia-mediated acidic pH reduce the amount of NO produced by NO synthase and enhance its ability to uncouple and generate mathematical equations. Because nitrites compete with xanthine at the Mo-co site and can be converted to NO, these conditions increase affinity for nitrites while decreasing XOR affinity for xanthine. The number of mathematical equations formed by XOR is sufficient to react with NO and produce RNS, especially ONOO^−^, under the same conditions [85].

### 4.6. NO Synthase and ROS Generation

The NO synthase (NOS; EC 1.14.13.39) produces NO, one of the smallest and most important signaling molecules in biological systems. Three major isoforms exist—neuronal (nNOS), inducible (iNOS), and endothelial (eNOS)—which differ in tissue distribution, regulation, and Ca^2+^ distribution. All isoforms catalyze the oxidation of L-arginine to L-citruline and NO using molecular O_2_ as a substrate and require multiple cofactors, including (6R-)5,6,7,8-tetrahydrobiopterin (BH_4_), reduced nicotinamide-adenine-dinucleotide phosphate (NADPH), flavin adenine dinucleotide (FAD), and flavin mononucleotide (FMN) [86]. During catalysis, electrons are transferred from NADPH through the flavins FAD and FMN in the reductase domain, where L-arginine is oxidized. BH_4_ plays a critical role in maintaining NOS dimer stability and facilitating efficient electron transfer. However, when substrate and cofactors are limited, NOS becomes uncoupled and generates O_2_^•−^ instead of NO [87].

This dysfunction is especially relevant for eNOS, where aging and pathological conditions promote uncoupling through mechanisms such as BH_4_ depletion, increased endogenous eNOS inhibitor asymmetric dimethylarginine (ADMA), and shortage of L-arginine. The resulting O_2_^•−^ reacts with NO, forming ONOO^−^, further oxidizing BH_4_ to BH_2_ and perpetuating eNOS dysfunction. In addition, peroxynitrite enhances L-arginine export and reduces its endothelial uptake, further limiting NO production [37]. Disrupted NOS function contributes to excessive ROS and reactive nitrogen species formation, promoting redox imbalance and cellular injury. In the nervous system, mitochondrial oxidative stress and altered NO signaling have been associated with neuronal loss and cell death pathways, including apoptosis and pyroptosis, in disorders such as epilepsy [88].

### 4.7. Endoplasmic Reticulum and ROS

The endoplasmic reticulum (ER) is a dynamic tubular network responsible for the synthesis, folding, and post-translational modification of transmembrane and secretory proteins. Newly synthesized polypeptides enter the ER in an unfolded state, where they are properly folded and assembled; proteins that fail to attain correct conformation are retained for refolding or targeted for degradation. This quality control process is tightly regulated by the ER redox environment, which plays a critical role in determining the fate of incoming proteins. ER redox homeostasis is closely linked to ROS generation and is modulated by several redox-active mediators, including Ca^2+^, glutathione (GSH)/glutathione disulfide (GSSG), protein disulfide isomerase (PDI)-endoplasmic reticulum oxidoreductin (ERO)-1, NADPH oxidase 4 (Nox4), and NADPH-P450 reductase (NPR). Disruption of this balance leads to ER stress, which is characterized by the accumulation of unfolded or misfolded proteins. Various stressors, such as high temperatures, salt, and pathogen infection, can impair protein-folding capacity and increase the burden on the ER quality control system, ultimately promoting ROS production and activation of stress signaling pathways [89].

Since ER oxirreductin-1 (ERO1) is the primary source of cellular ROS, the ER in eukaryotic cells is primarily responsible for producing them. A normal level of ROS is necessary for the formation of disulfide bonds, which is a crucial step in the machinery that folds proteins [37]. The most prominent and well-preserved ER stress sensor is the transmembrane protein inositol-requiring kinase 1 (IRE1), which has an RNAase domain in the cytosolic region and a Ser/Thr kinase domain [90]. Through the cleavage of microRNAs, the RNAase function of IRE1 is connected to the inflammatory signaling pathways of the inflammasome NRLP3 [91]. Additionally, the kinase component of IRE1 can activate the tumor necrosis factor α (TNFα) receptor-associated factor 2 (TRAF2), which, in turn, can activate the c-Jun N-terminal kinase (JNK) and NF-κB pathways [92]. These mechanisms are directly linked to the maintenance and growth of ROS production in cells [93]. Furthermore, ROS generation may be triggered by the ER’s release of Ca^2+^ [94]. The Ryanodine Receptor (RyR), a Ca^2+^ channel found in the ER, is regulated by the redox-sensing thiol groups. RyR can be activated by oxidation of its thiol sites, which allows the ER to release Ca^2+^ [95]. The two main biological sources of ROS, mitochondrial failure and NADPH oxidase hyperactivity, are intimately linked to dysregulated Ca^2+^ signaling [96]. Additionally, the ER expresses NOX4, one of the NADPH oxidase isoforms that feeds the ER’s vicious cycle of oxidative stress [97].

### 4.8. Cytochrome p450 and ROS Production

The mitochondrial electron transport chain and the cytochrome P450 (CYP)-dependent microsomal electron transport system are major endogenous sources of ROS. CYP enzymes are a diverse family of heme-containing monooxygenases widely expressed throughout the body and play a central role in phase I metabolism. They catalyze the oxygenation of organic substrates using molecular oxygen and are involved in the biotransformation of drugs, xenobiotics, and endogenous compounds such as sterols, fatty acids, eicosanoids, and vitamins [98,99].

During CYP-catalyzed monooxygenation, molecular oxygen binds to the enzyme–substrate complex, forming an activated oxo intermediate [100]. Electrons supplied by NADPH reduce this complex, generating a peroxide intermediate that undergoes protonation to release water and produce a highly reactive species capable of abstracting hydrogen atoms from the substrate. This leads to substrate oxidation, after which the product is released, and the enzyme returns to its resting state [101].

However, when electron transfer and oxygen activation become inefficient (a process known as uncoupling), oxygen is only partially reduced, leading to the formation of ROS such as O_2_^•−^ and H_2_O_2_. The accumulation of these species during xenobiotic metabolism contributes to oxidative stress and cytotoxicity [102].

ROS are continuously produced due to poor coupling of the P450 catalytic cycle, which affects signaling pathways and other cellular processes. P450-mediated ROS production is tightly regulated by transcriptional control and protein–protein interactions, which influence enzyme stability and coupling. The management of gene transcription and the modification of interactions between the monooxygenase’s protein constituents, which impact its activity, coupling, and stability, tightly govern the P450-mediated production of ROS. Lipid peroxidation and oxidative stress may arise from a surge in ROS generation brought on by dysfunction of these systems. P450 levels are subsequently downregulated by oxidative stress via multiple feedback mechanisms [103].

### 4.9. Peroxidases and ROS Production

Peroxidases are a class of enzymes involved in the detoxification of cellular peroxide radicals. In mammals, the prototypes are catalase (CAT) and glutathione peroxidases (GPx) [104]. CAT, a peroxisomal enzyme with a high turnover rate and strong substrate specificity, rapidly decomposes hydrogen peroxide (H_2_O_2_) into water and molecular oxygen, thereby limiting oxidative damage and preventing the formation of more reactive species such as hydroxyl radicals and peroxynitrite. Although H_2_O_2_ is potentially harmful at elevated concentrations, it also functions as an important signaling molecule regulating physiological processes such as cell proliferation, apoptosis, and immune responses. Therefore, tight regulation of H_2_O_2_ levels by antioxidant enzymes like CAT is essential for maintaining cellular redox balance [105].

Under certain conditions, CAT may also contribute to ROS formation. Some bacterial CAT (hydroperoxidases), including *Escherichia coli* HP-I and HP-II, generate ROS upon exposure to ultraviolet B (UVB) radiation. Notably, only HP-I, which contains NADPH as a cofactor, exhibits this activity, suggesting a regulatory role of NADPH. Structural studies of mammalian catalase show that NADPH is bound within a confined domain, and UVB-induced oxidation of NADPH can trigger conformational changes that enhance ROS formation. These findings indicate that NADPH oxidation may influence the dual role of CAT as both an antioxidant enzyme and a potential ROS source under photo-oxidative stress [106].

GPx (EC 1.11.1.9) are used by any oxygen-consuming eukaryotic cell to balance the production and recycling of either harmful or physiological oxygen byproducts. GPx focuses on the minor physiological adjustment of H_2_O_2_ concentrations in the extracellular and intracellular spaces. Because GPx can recycle organic peroxidized molecules, such as those found in free polyunsaturated fatty acids (PUFA) and complex membranes like phospholipid hydroperoxides, in addition to H_2_O_2_, it is more adaptable than CAT in terms of the substrates they can metabolize. As a result, they function as both scavengers and repairing enzymes. Therefore, GPx is thought to be the primary regulator of H_2_O_2_ concentration and, in turn, of H_2_O_2_-mediated assaults in and surrounding the majority of cells, despite CAT being a potent H_2_O_2_-recycling enzyme [107].

On the other hand, interactions between halide ions and H_2_O_2_ are mediated by haloperoxidases, another class of peroxidases. Three enzymes from this class have been found in mammals: eosinophil peroxidase, myeloperoxidase (MPO), and lactoperoxidase. By oxidizing iodide, bromide, and chloride ions, these enzymes can generate reactive halogen species such as HOCl, which enhance immunity and aid in the removal of many pathogens. However, when it persists, it causes oxidative stress [37].

### 4.10. Cyclooxygenase and ROS Production

Prostaglandin (PG) biosynthesis from arachidonic acid (AA) is catalyzed by cyclooxygenase (COX), which is the bifunctional heme-containing enzyme COX. It is a bifunctional enzyme that demonstrates peroxidase and cyclooxygenase activity. It adds two oxygen molecules to AA to create PGG2, a cyclic hydroperoxy endoperoxide. Peroxidase then reduces it to produce PGH2, a hydroxy endoperoxide [108]. COX-2 activity plays a significant role in the production of ROS and oxidative stress after ischemic injury [109,110]. Additionally, enhanced COX-2 activity is linked to increased O_2_^•−^ production, which exacerbates oxidative imbalance in neural tissue [111,112]. Moreover, COX-2 can generate carbon-centered radicals, which can lead to lipid peroxidation and the production of dopamine quinones, both of which are harmful to neurons [113,114]. A study concluded that COX-2 and ROS mutually amplify each other, driving vascular dysfunction in hypertension, and inhibiting COX-2 or NADPH oxidases restores redox balance and normal vascular tone [115].

## 5. ROS as Signaling Molecules

**Figure 4:**
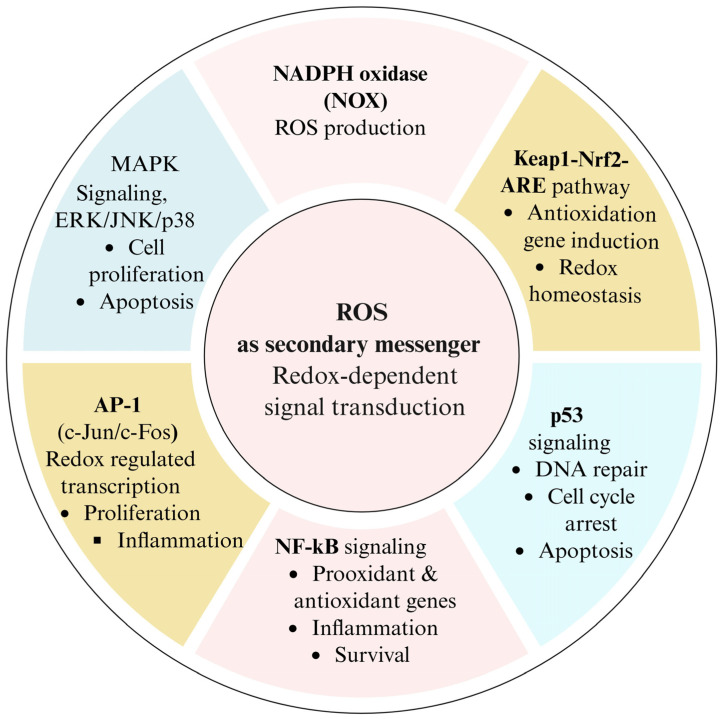
ROS-mediated redox signaling and regulation of cellular fate. Schematic representation showing ROS acting as secondary messengers that regulate key redox-sensitive pathways, including Keap1–Nrf2–ARE (antioxidant defense and redox homeostasis), NF-κB (inflammation and cell survival), MAPK/ERK/JNK/p38 and AP-1 (cell proliferation, differentiation, and stress responses), p53 signaling (cell cycle arrest and apoptosis), and NADPH oxidase (major ROS source). Dysregulation of these interconnected nodes contributes to the pathogenesis of major disorders, where impaired Nrf2 signaling is linked to metabolic and neurodegenerative diseases, persistent NF-κB and MAPK/AP-1 activation promotes chronic inflammation and cancer progression, altered p53 responses influence tumor development, and NOX-derived ROS drive vascular, renal, and pulmonary pathologies. Created in BioRender. Rahmani, AH. (2026) https://app.biorender.com/illustrations/canvas-beta/699a1d98be8affa1e25a5770, accessed on 6 March 2026.

Despite their potentially harmful effects, ROS have recently been shown to play a dual role as essential signaling molecules that regulate a wide range of biological processes (Figure 4) [116]. ROS are crucial signaling molecules at the physiological level that support a wide range of cellular processes, such as immune response, stress adaptation, cell growth, proliferation, differentiation, and apoptosis [117]. By modifying the activity of several enzymes, transcription factors, and signaling cascades, ROS serve as secondary messengers [116]. Thus, knowledge of ROS’s dual nature as hazardous and regulatory molecules is crucial for comprehending the intricate interactions among oxidative stress, cellular metabolism, and biological process regulation. Recently, researchers uncovered several key signaling pathways and transcription factors that respond to ROS, highlighting how closely their activity depends on ROS regulation [15].

According to recent research, ROS (mitochondrial, cytosolic, and nuclear) that are specific to different compartments variably activate transcription factors, including Nrf2, NF-κB, and AP-1, resulting in varied outcomes like senescence, apoptosis, and proliferation [15]. The dynamics of ROS may be precisely monitored because of developments in detecting technologies, including biosensors, redox-sensitive probes, and proteomics [3,19]. Redox alterations are linked to gene expression by ROS-driven post-translational modifications, which have direct implications for disease: they exacerbate diabetic vascular injury, cause neuronal loss in neurodegeneration, and promote cancer chemoresistance.

The following sections describe these ROS-responsive pathways and explain how they connect ROS dynamics with alterations in gene expression. Table 3 summarizes key redox-sensitive signaling pathways activated by specific reactive oxygen species (ROS). It highlights primary molecular targets, downstream transcriptional regulators, and the resulting cellular outcomes, including stress adaptation, inflammation, proliferation, apoptosis, senescence, and antioxidant responses. ROS, while potentially harmful, also act as essential signaling molecules regulating processes like immune response, cell growth, and apoptosis. Their dual role influences key enzymes, transcription factors, and signaling pathways, linking oxidative stress to cellular metabolism and biological regulation (Figure 4).

### 5.1. MAPK Signaling Pathway

Mitogen-activated protein kinases (MAPKs) are a family of serine/threonine protein kinases that play a central role in transmitting signals from the cell surface to the nucleus. The three-kinase signaling module is made up of the MAPK, a MAPK kinase (MAP2K), and a MAPK kinase (MAP3K). MAPKs include growth factor-regulated extracellular signal-related kinases (ERKs), stress-activated MAPKs, c-jun NH2-terminal kinases (JNKs), and p38 MAPKs. MAPKs are activated when MAP2Ks are phosphorylated by MAP3Ks. Although the exact mechanisms by which ROS can activate ERKs, JNKs, or p38 MAPKs are unknown, increased ROS production in a cell typically results in their activation [132]. Activation of p38 MAP kinase enhanced ROS production, which could subsequently promote cell death via a variety of mechanisms including mitochondrial dysfunction [118]. H_2_O_2_ triggers ROS-activated p38 MAPK signaling, which inhibits malignant transformation and causes cell cycle arrest by suppressing oncogenic H-Ras activity [119].

### 5.2. Activator Protein-1 (AP-1) Transcription Factor (TF) Family

ATF, c-JUN, and c-FOS are among the members of the Activator Protein-1 (AP-1) transcription factor (TF) family, which mediates several biological processes, including cell death, differentiation, and proliferation. The role of AP-1 TFs in cancer development has been thoroughly examined since their discovery. The intricacy of these TFs has been brought to light by numerous in vitro and in vivo investigations, primarily because of their cell-type-specific homo- or heterodimerization, which produces a variety of transcriptional response profiles. However, AP-1 TFs are becoming recognized as promising therapeutic targets for a variety of malignancies due to a growing understanding of their role in disease [133].

It is redox-regulated, and extracellular stimuli that activate AP-1 are dependent on ROS. For instance, exogenous ROS exposure increases c-Fos and c-Jun gene expression and protein levels, thereby strengthening the cells’ ability to bind DNA. Thus, ROS scavengers or antioxidants can effectively prevent UVB- or AP-1-mediated activation by carcinogenic chemicals. The redox state of a number of conserved cysteine residues determines the DNA-binding activity of c-Fos and c-Jun [15]. Previous reports showed that inhibition of AP-1 coincided with decreased ROS production [134]. In contrast, polyunsaturated fatty acids (PUFA) induce intracellular oxidative stress and activate AP-1 in human fibroblasts [135].

### 5.3. Keap1-Nrf2-ARE Pathway

The NRF2/KEAP1 signaling pathway represents a primary cellular defense mechanism against oxidative stress and plays a central role in maintaining redox homeostasis under basal conditions. Kelch-like ECH-associated protein 1 (Keap1), a component of Curlin-dependent E3 ubiquitin ligase complex, binds to NRF2 and promotes its ubiquitination and proteasomal degradation, thereby tightly regulating NRF2 activity. Many Nrf2 inducers easily interact with cysteine thiol groups of Keap1’s, leading to conformational changes that disrupt the Keap1-Nrf2 complex and stabilize NRF2 [136]. Recent evidence suggests that KEAP1 functions as a multifunctional stress sensor, integrating signals from oxidative stress, cellular metabolites, and inactivation mechanisms and impaired autophagy to modulate NRF2 activation [137].

Nuclear factor-erythroid 2-related factor 2 (Nrf2) is a chief regulator of antioxidant defense and cellular redox balance. Upon activation, it translocates to the nucleus and binds to antioxidant-response elements in the promoter region of targeted genes, leading to transcriptional up-regulation of multiple cytoprotective proteins. These include heme oxygenase-1 (HO-1), NADPH:quinone oxidoreductase 1 (NQO1), glutathione reductase, thioredoxin reductase, superoxide dismutase (SOD), glutathione peroxidase (GPx), ferritin, and glutathione S-transferase (GST) [138,139,140].

### 5.4. NF-kB

Nuclear factor K-light-chain-enhancer of activated B cells, or NF-κB, is a family of transcription factors that plays a central role in regulating immune and pro-inflammatory responses. In resting cells, NF-κB remains sequestered in the cytoplasm through its interaction with inhibitory proteins of the IκB family. Upon stimulation, IκB is phosphorylated by IκB kinase complex and subsequently degraded via the proteasome, allowing NF-κB to translocate into the nucleus where it promotes the transcription of genes involved in inflammation, immune regulation, cell survival, and proliferation [141].

The NF-κB family consists of NF-κB1, NF-κB2, p65 (RelA), c-Rel, and RelB. All NF-κB proteins have a Rel-homology (RHD) domain, required for DNA binding and dimerization. Activation of the canonical NF-κB pathway typically occurs through stimulation of pro-inflammatory receptors, such as those belonging to the tumor necrosis factor (TNF) receptor superfamily, leading to nuclear translocation of NF-κB protein dimers and regulation of target gene expression [142].

ROS have a dual and context-dependent role in NF-κB signaling [25]. Moderate ROS levels can function as signaling molecules that activate NF-κB by modifying the IκB kinase (IKK) complex, resulting in IκB phosphorylation and degradation. Conversely, excessive oxidative stress or antioxidant treatment can suppress NF-κB activation [25,143]. Early studies demonstrated that micromolar concentrations of H_2_O_2_ activate NF-κB in human T cells, an effect inhibited by the antioxidant N-acetyl cysteine, suggesting a redox-sensitive regulatory mechanism [144]. However, later evidence indicates that H_2_O_2_ may act more as a modulator than a direct inducer of NF-κB signaling [123].

In addition, ROS-induced DNA damage can indirectly activate NF-κB. DNA damage sensors such as ATM and ATR kinases phosphorylate the NF-κB essential modulator (NEMO), a key regulatory component of the IKK complex. This process links genotoxic stress to inflammatory signaling by promoting IKK activation and subsequent NF-κB nuclear translocation [145].

### 5.5. p53 Signaling

Tumor suppressor protein p53 is a redox-sensitive transcription factor that plays a central role in coordinating cellular stress responses. Wild-type p53 regulates the transcription of numerous genes and directs cells toward cell cycle arrest, senescence, or apoptosis, thereby preventing the propagation of damaged DNA. One of the key challenges in p53 biology is understanding how it selectively regulates specific sets of target genes to determine a particular cellular outcome (such as favoring cell cycle arrest over senescence or apoptosis). This selectivity is influenced by multiple modulators, including regulatory proteins and non-coding RNAs (such as myc, hCAS/CSE1L, Hzf, and miR-34), which contribute to the differential transactivation of p53 target genes and ultimately shape cell fate decisions [146].

ROS function as important upstream signals that activate p53 in response to cellular stress. Upon activation, p53 can induce the transcription of several antioxidant genes, including manganese SOD and GPX1, helping to regulate redox balance and influence tumor cell survival. ROS can also function as a p53 downstream factor to promote ferroptosis and apoptosis in tumor cells [147].

At the same time, ROS can act as signaling molecules to initiate p53 activation. Genotoxic stressors, including ionizing radiation and chemotherapeutic agents, increase intracellular ROS levels, which can oxidize specific cysteine residues within p53. These redox modifications induce conformational changes that stabilize and activate the protein. Activated p53 then translocates to the nucleus, where it regulates the expression of genes involved in DNA repair, cell cycle arrest, and apoptosis, thereby maintaining genomic integrity [15].

**Figure 5:**
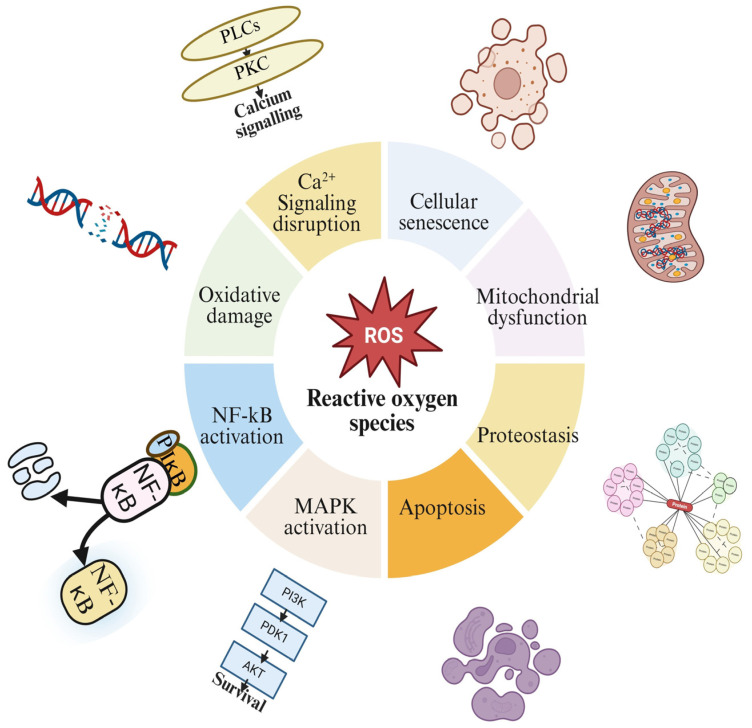
Dual role of reactive oxygen species in cellular signaling and stress responses. By controlling the MAPK and NF-κB pathways, reactive oxygen species (ROS) serve as physiological signaling mediators that promote immunological responses, tissue repair, and regulated cell proliferation. Excess ROS cause mitochondrial dysfunction, oxidative macromolecular damage, Ca^2+^ imbalance, and compromised proteostasis when oxidative stress is prolonged. Major pathologies such as cancer (enhanced proliferation and survival signaling), neurodegenerative diseases (mitochondrial dysfunction and proteostasis failure), cardiovascular and metabolic disorders (Ca^2+^ dysregulation and NF-κB-driven inflammation), and aging-related tissue degeneration are mechanistically linked to these changes, which promote apoptosis, cellular senescence, and abnormal proliferation. Created in BioRender. Rahmani, AH. (2026) https://app.biorender.com/illustrations/canvas-beta/6957b13ebd038604a1ebf3db, accessed on 5 March 2026.

Collectively, ROS act as central regulators of cellular signaling, organelle integrity, and cell fate decisions. A comprehensive overview of ROS-mediated cellular outcomes is summarized in Figure 5.

## 6. ROS-Mediated Toxicities

Cells continuously regulate ROS levels to maintain normal physiological functions. However, excessive ROS formation affects the antioxidant defense system and prevents its potential to eliminate excess free radicals because it lowers the activity of the antioxidant system as well as total antioxidant content. Hyperoxia, inflammation, and a weak or compromised antioxidant system all contribute to the production of excessive ROS, which ultimately disrupts the homeostasis of the biological system as a whole [148]. In general, ROS are reactive and can degrade into biological macromolecules such as proteins and DNA, causing oxidative stress that impairs cellular functions [149].

Because of their high abundance in cells, extracellular tissues, and bodily fluids, as well as their quick reaction rates with oxidants, proteins are essential targets for oxidation reactions. Furthermore, lipids and carbohydrates can be broken down by oxidative stress into extremely reactive intermediates that ultimately target proteins at different functional sites. As a result, protein oxidation, glycoxidation, and lipoxidation produce a wide range of unique post-translational protein modifications. Non-reversible changes may contribute to pathological conditions and various diseases, whereas reversible changes are important in physiological processes and form signaling mechanisms (also known as “redox signaling”) [7].

ROS can profoundly alter proteins, affect their functioning, and increase their ability to cause cellular damage. The attack of ROS on proteins leads to the formation of carbonyls by modifying amino acids such as lysine and glutamic acid. Ultimately, these proteins lose their normal shape and function due to ROS attack. ROS can also induce lipid oxidation, producing reactive aldehydes that attach to proteins through a process called lipoxidation, forming toxic byproducts known as advanced lipoxidation end products (ALEs) [150]. Oxidative modifications can occur in a number of amino acidic residues, such as through the chlorination of aromatic groups and primary amino groups, oxidation of sulfur-containing residues, nitration of tyrosine residues, hydroxylation of aromatic and aliphatic groups, nitrosylation and glutathionylation of cysteine residues, and conversion of certain amino acid residues to carbonyl derivatives [151,152].

Lipid peroxidation occurs when ROS attack membrane phospholipids or polyunsaturated fatty acids, leading to the formation of reactive compounds such as aldehydes, ketones, and peroxides. Among these, malondialdehyde (MDA) is a key toxic byproduct that damages macromolecules, including DNA and proteins, through oxidation, fragmentation, and crosslinking. These reactions disrupt membrane integrity, alter electrolyte balance, and impair cellular function. Lipid peroxidation proceeds as a chain reaction, initiated by ROS attack on polyunsaturated fatty acids to form carbon-centered radicals, followed by addition of oxygen, generating lipid peroxyl radicals and hydroperoxides. This amplifies oxidative stress and cellular damage [153].

In a hyperglycemic state, sugars react with the amino groups of proteins, forming advanced glycation end products (AGEs). These AGEs, like carboxylmethyl lysine (CML) and pentosidine, build up over time in long-lived proteins and are closely linked to diseases such as diabetes, atheroscelerosis, kidney failure, and Alzheimer’s disease. In essence, ROS-induced oxidation, lipioxidation, and glycation work together to damage proteins, disrupt cellular balance, and contribute to the progression of many chronic disorders [150].

By negatively altering proteins and other molecules, ROS, such as hydroxyl radicals and ^1^O_2_, act on DNA, disrupting cellular function. They alter DNA bases, break single and double strands, crosslink DNA with proteins, and damage the repair system [154]. ROS can attack DNA, causing base modifications, strand breaks, and adduct formation. For example, OH reacts with DNA bases and the sugar backbone and produces 7,8-dihydroxy-8-oxo-2′-deoxyguanosine (8-oxodG). DNA bases can be deaminated when exposed to reactive nitrogen species, and guanine can be converted into xanthine, oxanine, and 8-nitroguanine, which is quickly removed from DNA by spontaneous depurination. 5-chlorocytosine and 5-chlorouracil are the two main byproducts of HClO. These altered bases are a sign of HClO-mediated genomic base modification in vivo and have been found at inflammatory sites [155].

## 7. Approaches for Evaluation of ROS in Clinical and Preclinical Samples

Oxidative stress markers are crucial tools for evaluating the biological redox status, the state and course of disease, and the benefits of antioxidants for human health. Numerous studies have focused on identifying oxidative stress markers, and over the past few decades, several markers from various biomolecule sources have been proposed. However, there is disagreement over reproducibility, standardization, and validation for some of them [3,150,155].

ROS are challenging to measure in vivo because they exist at very low nanomolar steady-state concentrations due to their high reactivity and multiple clearance mechanisms [156]. To compete with antioxidants and generate stable, quantifiable products, ROS detection in biological systems requires probes that react quickly with ROS [157]. Because ROS are short-lived, extremely reactive, and challenging to measure directly, Murphy and colleagues advise a standardized approach for assessing ROS and oxidative damage. The recommendations place a strong emphasis on choosing techniques according to the particular kind, source, and biological context of ROS, as well as differentiating between direct ROS detection and indirect evaluation using oxidative damage biomarkers. It is frequently more convenient to measure end products such as F_2_-isoprostanes, 8-OHdG, and protein oxidation biomarkers in clinical and in vivo investigations; nevertheless, these indicate cumulative damage rather than current ROS levels. The authors highly recommend avoiding nonspecific assays like TBARS, total antioxidant capacity tests, and commercial kits with inadequate validation, as well as utilizing multiple validated biomarkers and validating results using complementary approaches [158].

**Figure 6:**
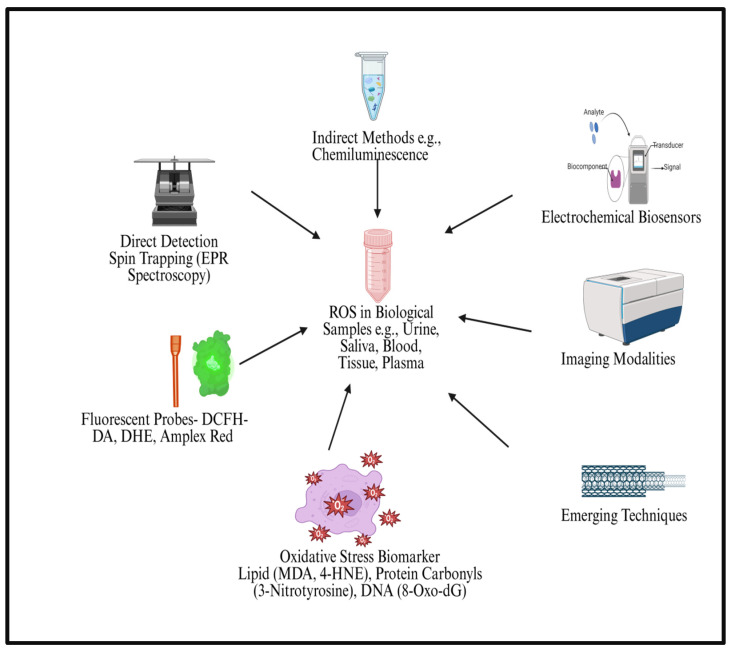
Approaches for detection and analysis of reactive oxygen species. The figure illustrates a workflow for reactive oxygen species (ROS) analysis, beginning with biological sample preparation and progressing through multiple complementary detection platforms. It highlights the use of biosensors, fluorescence-based probes, imaging systems, spectrophotometric assays, chemiluminescence, and chromatographic techniques for ROS quantification. Together, these methods provide a comprehensive assessment of ROS levels and oxidative stress in biological systems. Created in BioRender. Rahmani AH. (2026) https://app.biorender.com/illustrations/canvas-beta/6953c2971ad2e4b4c45f8921, accessed on 2 January 2026.

For the identification or measurement of ROS production, several direct and indirect techniques have been put forth [159] (Figure 6). Unfortunately, the concentration of free radicals involved in OS processes at toxic levels is so low that it is very difficult to detect or quantify these extremely short-lived species [160]. Across these detection platforms, a variety of specialized ligands are employed, including spin-trapping ligands such as DMPO and DEPMPO, oxidant-responsive fluorescent ligands such as DCFH-DA, dihydroethidium, and Amplex Red, chemiluminescent ligands such as luminol and lucigenin, and genetically encoded redox-sensitive ligands such as roGFP and HyPer [161]. Together, these ligands enhance the specificity, sensitivity, and spatial resolution of ROS measurements in both clinical and preclinical settings (Table 4). Figure 6 summarizes commonly used experimental approaches for detecting and analyzing reactive oxygen species (ROS) in biological samples.

### 7.1. Direct Measurement ROS

Electron paramagnetic resonance (EPR) spectroscopy is the gold standard for direct detection of ROS. EPR allows for the identification of specific free radicals by detecting unpaired electrons. Spin traps, or spin probes, are commonly used to stabilize transient ROS. Although this method has been applied to isolated tissues and experimental animal models, its clinical application is limited by high cost, specialized equipment, and difficulties with in vivo measurements [162]. Oxygen-, carbon-, nitrogen-, and sulfur-centered radicals can be measured using EPR spin trapping with either DMPO or DEPMPO. However, they have the drawback that spin adducts can be transformed into EPR-silent products when applied to tissues or cells, so low-level radical production cannot be ruled out if no signal is detected. High-performance liquid chromatography (HPLC), mass spectroscopy, high-field nuclear magnetic resonance, and antibody-based detection are alternative methods for detecting spin trap adducts or their metabolites [163].

### 7.2. Fluorescent Methods

Oxidant-sensitive fluorescent probes that are non-fluorescent before oxidation by ROS are used in fluorescence-based detection techniques. The most widely used candidates for fluorescent probe-based detection techniques are Amplex Red (impermeable to cells), dichlorodihydro fluorescein, and dihydroethidium. Cell-permeable techniques measure the generation of radicals during stimulation and assess the oxidative state of cellular compartments. These probes typically undergo oxidation through a one-electron free radical mechanism, producing probe radical intermediates and fluorescent products [164]. Small-molecule fluorescent probes are widely used in biology, pathology, and medical diagnostics due to their advantages of non-invasiveness, high sensitivity, and in vivo, real-time detection. For early disease diagnosis and assessment of therapeutic conditions, the improved use of small-molecule fluorescent probes to detect ROS levels in organisms is essential [165]. Although fluorescence imaging offers numerous benefits as a detection technique, creating effective probes for real-time detection poses several drawbacks. These include temperature, variations in concentration, ambient conditions, source light fluctuations, and instrument sensitivity [166].

Because of their great sensitivity, selectivity, and ease of use, fluorescence-based techniques are the most often used O_2_^•−^ detection methods for viewing and tracking O_2_^•−^ levels in live cells. These techniques depend on the interaction of oxidant-sensitive or non-redox fluorescent probes with O_2_^•−^ generated by cells, which leads to probe activation and light emission. Cellular sources of superoxide formation can be identified by measuring the ensuing fluorescence signal, which is frequently accompanied by a noticeable color shift, using fluorescence-based sensors [171]. Fluorescence-based methods enable sensitive detection of hydroxyl radicals (^•^OH) despite their short lifetime and low abundance. Advances such as NIR-II imaging, ratiometric probes, and mitochondria-targeted fluorescent sensors have improved selectivity and enabled non-invasive, in vivo, and subcellular visualization of ^•^OH [166].

### 7.3. Chemiluminescent Methods

Similar to fluorescence-based assays, chemiluminescence analysis (CLA) is frequently used to detect O_2_^•−^, due to its high sensitivity, ease of use, and lack of an excitation light source. Common CLA probes, such as lucigenin, luminol, and its counterpart L012, produce light when they react with reactive species, enabling rapid monitoring of oxidative activity in cells and biological systems. However, these probes often have limitations such as cellular antioxidant interference, redox cycling, nonspecific interactions with many reactive oxygen species, and potential false-positive findings. While CLA methods offer rapid, highly sensitive detection of low-level O_2_^•−^ generation, precise evaluation of O_2_^•−^ production requires careful interpretation and supplementary assays [164].

### 7.4. Electro-Chemical Biosensing (ECB)

By applying cytochrome c and polyaniline-sulfonic acid to gold wire electrodes, electrochemical biosensors (ECBs) enable sensitive and selective detection of O_2_^•−^. This method produces an electrical current proportional to the concentration of O_2_^•−^ by reducing redox-active proteins, which are then reoxidized at the electrode surface. In both in vitro and in vivo situations, ECB enables real-time monitoring of O_2_^•−^ production and its interactions with antioxidants. Compared with single-layer coatings, layer-by-layer protein immobilization increases sensitivity, and the addition of cytochrome c improves detection selectivity. Nevertheless, the availability of appropriate redox proteins for electrode modification restricts the procedure [164].

### 7.5. Biomarkers of Oxidative Stress

Biomarkers of oxidative stress are widely used in clinical and preclinical studies because ROS and RNA are highly short-lived and difficult to measure directly. Instead, stable end products of oxidative damage to lipids, proteins, and nucleic acids serve as reliable biomarkers of redox imbalance. Among lipid peroxidation markers, malondialdehyde (MDA) and 4-hydroxynonena (4-HNE) are commonly quantified in plasma, serum, urine, or tissues using spectrophotometric assays, ELISA, HPLC, or mass spectrometry. These markers reflect oxidative damage to cellular membranes and lipoproteins and are widely applied due to their relative stability and accessibility, although assay specificity and methodological standardization remain important considerations [150].

Protein oxidation markers provide complementary information on oxidative damage to structural and functional proteins. Protein carbonyls are essential biomarkers of protein oxidation and are produced by numerous oxidative and glycoxidative mechanisms. DNPH-based spectrophotometric, ELISA, immunoblotting, or MS-based enrichment techniques are frequently used to quantify carbonyls, which rise with aging and disease. Each technique has unique advantages and disadvantages [7]. Although immunological techniques are highly sensitive for detecting protein oxidation, they suffer from limited specificity, quantification, and epitope accessibility, necessitating meticulous validation. On the other hand, certain aromatic amino acid oxidation products (such as 3-chlorotyrosine, 3-bromotyrosine, and 3-nitrotyrosine) can be measured using LC-MS, HPLC, electrochemical, or immunoassays, providing greater insight into the type of oxidant involved. In general, rather than relying on a single biomarker, accurate evaluation of protein oxidation requires combining several analytical techniques [7]. The most popular method for evaluating oxidative damage to nucleic acids is to measure 8-oxodG, a well-known indicator of oxidative DNA damage associated with mutagenesis, aging, and cancer risk [170]. ELISA, HPLC-ECD, or LC-MS/MS can be used to find 8-oxodG. The current agreement highlights that no single biomarker is sufficient, as oxidative stress affects multiple biomolecular targets simultaneously. In clinical and preclinical research, an integrated panel of lipid, protein, and DNA oxidation markers—ideally in conjunction with antioxidant defense indices—offers a more thorough and biologically significant evaluation of oxidative stress [150].

### 7.6. Imaging Modalities for ROS

Despite the significance of imaging modalities, accurately detecting oxidative stress and ROS-associated diseases remains challenging due to the short duration, low concentration, and uneven spatial distribution of ROS within tissues. Magnetic resonance imaging (MRI) provides superior depth and spatial resolution and can indirectly represent oxidative stress through relaxation changes, despite its cost and lack of real-time ROS tracking. Despite being widely accessible and suitable for deep tissues, ultrasound has a limited ability to detect ROS directly and is operator-dependent [172]. By stimulating fluorescent molecules and using high-sensitivity cameras to detect the signals that are released, fluorescence imaging employs light to see biological structures [173,174].

In ROS-targeted research, this method frequently uses near-infrared (NIR) light to activate fluorophores or contrast agents. Weak light penetration limits fluorescence imaging to superficial tissues, but when combined with ROS-responsive probes, it can detect ROS in real time and with high sensitivity. Photoacoustic imaging combines optical contrast with ultrasonic depth to improve sensitivity and spatial resolution for ROS mapping. In contrast, Raman imaging provides detailed molecular insights into oxidative damage but has a weak signal. Combining specific ROS-sensitive materials with complementary imaging techniques is a promising strategy for precise oxidative stress measurement in clinical and preclinical contexts [172].

### 7.7. Emerging Technologies for ROS Detection

Recent developments in microfluidic platforms, genetically encoded redox probes, and nanotechnology-based sensors have improved the sensitivity and specificity of ROS detection. Genetically encoded probes such as roGFP and HyPer allow real-time monitoring of redox changes in specific cellular compartments in preclinical models. Combining ROS measurements with antioxidant enzyme activity (such as GPx, CAT, and SOD) can yield a more comprehensive assessment of redox homeostasis [167,168]. Nanoparticle-based sensors are widely used for ROS detection due to their high stability, tunable surface chemistry, and efficient cellular targeting, including tumor accumulation through the increased permeability and retention effect. NPs can reduce nonspecific interactions and probe degradation while enabling protected, ratiometric, and more selective ROS readings by encasing ROS-sensitive dyes and reference probes. Additionally, some NPs—such as quantum dots or gold nanoparticles—have intrinsic optical properties that enable sensitive ROS detection without the need for external fluorophores, making them valuable tools for monitoring oxidative stress in cells and in vivo [169].

Although numerous sophisticated techniques have been developed for detecting reactive oxygen species, many are primarily suited to control experimental systems rather than direct clinical use. Due to their high reactivity, extremely low steady-state levels, and short half-life, accurate in vivo measurement of ROS in humans remains technically difficult. As highlighted in the recent literature, current detection strategies are largely limited to specialized approaches such as electron paramagnetic resonance, fluorescence-based probes, and molecular sensors, which are more feasible in preclinical settings. Consequently, clinical assessment of oxidative stress relies predominantly on indirect indicators, including stable oxidation products of lipids, proteins, and nucleic acids, which provide a more practical and reliable reflection of oxidative status in human disease [156,157,175].

## 8. Oxidative Stress in the Pathogenesis of Various Diseases

**Figure 7:**
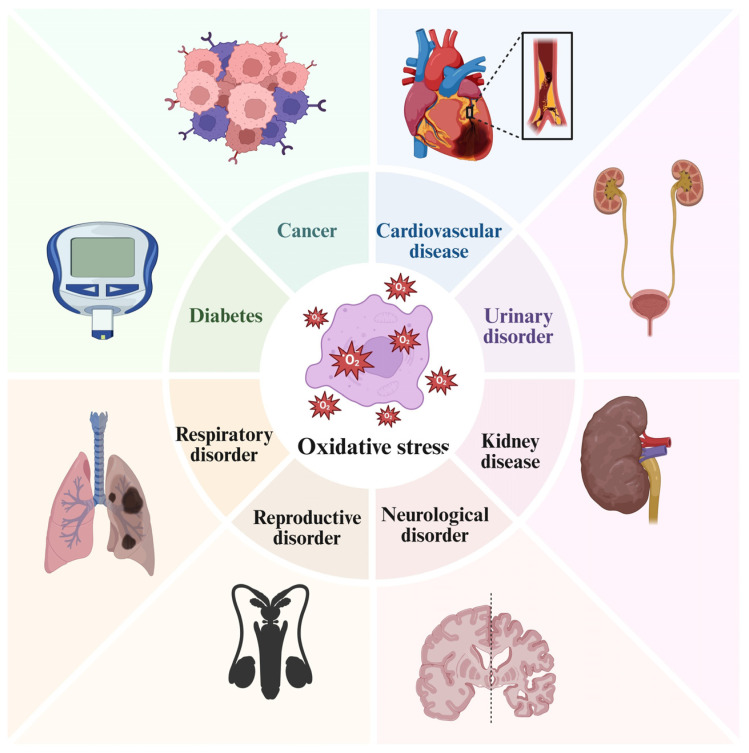
Central role of oxidative stress in the pathogenesis of multiple diseases. The figure illustrates oxidative stress as a central pathological driver linking excessive ROS production to multiple chronic diseases. Elevated ROS disrupt cellular homeostasis and contribute to the development of cancer, cardiovascular, neurodegenerative, respiratory, renal, metabolic, and reproductive disorders. Together, it highlights oxidative stress as a shared mechanistic hub underlying diverse disease pathophysiology. Created in BioRender. Rahmani AH. (2026) https://app.biorender.com/illustrations/canvas-beta/69aa7c7d0a6712a557b39daf, accessed on 6 March 2026.

Oxidative stress is now recognized as a major pathogenic factor in a number of neurological, vascular, and metabolic disorders (Figure 7). Under normal physiological conditions, ROS and RNS are essential modulators of intracellular signaling, metabolic regulation, and host defense. However, when their synthesis surpasses the antioxidant systems’ buffering capacity, a persistent redox imbalance develops, causing structural and functional damage to proteins, lipids, and nucleic acids. This oxidative damage is crucial for the onset and advancement of diabetes and obesity, in addition to diabetes-related microvascular consequences like diabetic retinopathy, nephropathy, atherosclerosis, and cardiovascular disease [176].

### 8.1. Oxidative Stress in Cancer

Cancer remains the leading cause of mortality worldwide, arising from a complex interplay of environmental exposures and genetic susceptibility. ROS play a dual and context-dependent role in cancer development. At moderate levels, ROS can promote tumor initiation, transformation, and proliferation, whereas at high concentrations they induce oxidative damage and trigger cell death [177,178]. During carcinogenesis, tumor cells reprogram sulfur-based metabolism, increasing NADPH production and activating antioxidant transcription factor. Genetic alterations that occur during early tumorigenesis enhance the ability of cells to survive oxidative stress, often through activation of PPP, AMPK signaling, and reductive glutamine and folate metabolism to maintain redox balance [179].

Treatment and prevention of carcinogenesis need to counteract oxidative stress and strengthen the antioxidant defense system [180]. Oxidative stress contributes to cancer progression by inducing mutations in important genes, altering signaling pathways, and impairing normal cellular functions. It promotes angiogenesis, uncontrolled proliferation, metastasis, and resistance to apoptosis [177]. ROS can disrupt tumor suppressor gene activity while simultaneously activating oncogenic signaling pathways, thereby facilitating tumor growth and survival [181]. In addition, chronic oxidative stress contributes to genomic instability, dysregulated cellular signaling, and compromised antioxidant defense systems [182].

Processes such as epithelial–mesenchymal transition (EMT) and abnormal angiogenesis are also influenced by elevated oxidative stress and play key roles in malignant transformation [183]. Clinical and experimental evidence supports the involvement of oxidative imbalance in multiple cancers. In colorectal cancer (CRC), patients exhibit increased total oxidant status and oxidative stress index along with altered antioxidant profiles. Elevated levels of oxidative damage markers, including oxidized glutathione, 8-oxo-2′-deoxyguanosine, and F2-isoprostanes, have been reported, while antioxidant enzymes such as catalase and reduced glutathione are often decreased. Similarly, in hepatitis B-related liver cancer, higher levels of malondialdehyde and uric acid, and lower levels of glutathione and superoxide dismutase are associated with postoperative recurrence. In hepatocellular carcinoma, tumor resection is often followed by reduced lipid peroxidation and improved antioxidant enzyme activity, indicating a dynamic relationship between oxidative stress and tumor burden [184,185,186,187].

In renal cell carcinoma, elevated oxidative stress and reduced antioxidant levels are associated with reduced antioxidant defenses and elevated prolidase activity [188]. In lung cancer, oxidative stress tends to increase as the disease progresses, while antioxidant enzyme levels decline. However, chemotherapy may partially restore redox balance in responsive patients [189]. In esophageal carcinoma, integrated analyses have identified oxidative stress and ER stress-related genes involved in pathways such as NOTCH signaling and oxidative stress-induced senescence [190]. Head and neck cancer patients also demonstrated increased oxidative stress indices and reduced antioxidant capacity, which may contribute to DNA damage and disease severity [191]. At the molecular level, oxidative stress affects not only DNA but also lipids and proteins. Lipid peroxidation generates reactive aldehydes such as crotonaldehydes, acrolein, 4-hydroxy-2-nonenal, and MDA, which harm DNA by forming exocyclic adducts. Mutations in key regulators of oxidative stress responses, including Kelch-like ECH-associated protein 1 (KEAP1) and nuclear factor erythroid 2-related factor 2 (NFE2L2), have been observed in certain cancers, particularly in HPV-negative head and neck cancer [192].

Oxidative stress is closely associated with the development and progression of colorectal cancer (CRC) [193]. Elevated inflammation and lower antioxidant defenses (CAT, GSH) have been associated with CRC [185]. Similar redox imbalance contributes to breast cancer pathogenesis [194]. In papillary thyroid cancer patients, increased oxidative DNA/RNA damage, enhanced lipid peroxidation, and decreased antioxidant capacity and SIRT3 levels are linked to tumor aggressiveness and metastasis [195].

ROS-induced toxicity contributes to cancer development through oxidative DNA damage, genomic instability, and redox-dependent activation of carcinogenic signaling pathways. At moderate concentrations, ROS activate key survival and stress-responsive pathways including p38, JNK, PI3K/Akt, MAPK/ERK1/2, and NF-κB, MMPs, VEGF, promoting cancer cell proliferation, angiogenesis, and survival [196]. ROS also promote EMT, tumor growth, and metastasis by stimulating MAPK, PI3K/Akt, JAK/STAT, Wnt/β-catenin, and TGF-β signaling. Additionally, they drive invasion and microenvironment remodeling [27]. Furthermore, hypoxia-associated ROS and mutant p53 activation further enhance VEGF production, thereby supporting tumor migration, proliferation, and metastatic progression [196].

### 8.2. Diabetes and Oxidative Stress

Oxidative stress plays a key role in the development and progression of diabetes mellitus (DM), influencing both disease onset and long-term complications. Early metabolic disturbances, including impaired glucose tolerance and obesity, promote insulin resistance, β-cell dysfunction, and cellular oxidative stress through lipid accumulation, altered trophic factor release, and disrupted signaling pathways [197].

Increased ROS production from mitochondrial electron transport chain, endoplasmic reticulum, phagocytic cells, and peroxisomes induces structural and functional damage to proteins, lipids, and nucleic acids, while altering several intracellular signaling pathways and impairing insulin action [198]. Hyperglycemia-induced ROS activates several major pathogenic pathways, including increased polyol pathway flux, enhanced formation of advanced glycation end products (AGEs), activation of protein kinase C (PKC) isoforms, up-regulation of AGE receptors and their ligands, and overactivity of the hexosamine pathway [199].

These processes promote inflammation, cellular dysfunction, and apoptosis, contributing significantly to both microvascular and macrovascular diabetic complications [200]. Importantly, oxidative damage markers such as 8-oxodG and 8-iso-PGF2α are elevated even at the prediabetic stage, supporting oxidative stress as an early contributor and potential biomarker for disease progression [201]. Elevated total oxidant status and reduced antioxidant capacity in prediabetes further reinforce its role as an early pathogenic factor and therapeutic target [202,203].

Oxidative stress also accelerates diabetes pathogenesis by impairing insulin production, promoting β-cell apoptosis, sustaining hyperglycemic memory, and triggering systemic inflammation. Excess ROS damage cellular macromolecules and reduce β-cell mass, while activation of NF-κB further contributes to β-cell death [13]. Additionally, oxidative stress disrupts insulin signaling by altering IRS-1/IRS-2, Akt, AMPK, mTOR, IKK-β, and p38 MAPK pathways, and suppresses key transcription factors such as PDX-1, MAFA, PPAR-γ, C/EBPs, MEF2, HIF-1α, and NF-κB, leading to reduced GLUT-4 expression and impaired glucose uptake. These changes collectively promote insulin resistance, mitochondrial dysfunction, and chronic inflammation, driving the transition from prediabetes to overt T2DM and its complications [204].

### 8.3. Neurodegenerative Disorders and Oxidative Stress

Oxidative stress is closely linked to the deposition of aberrantly aggregated proteins and the disturbance of metal ion balance. Neurodegenerative diseases are pathologically characterized by increasing cell malfunction and death that often affects a particular brain system, and are clinically characterized by their subtle onset and persistent development. The morphological hallmarks of many neurodegenerative diseases include neuronal loss linked to gliosis and, more often, protein misfolding and aggregation that results in the persistent buildup of aberrant extracellular and intracellular filamentous deposits in particular cell types. Selective neuronal susceptibility is a phenomenon in neurodegenerative disorders when certain populations of neurons are susceptible to increased oxidative stress, even though many brain neurons can withstand an increase in oxidative stress [205].

ROS-sensitive pathways come together to cause gradual neuronal death through mitochondrial damage, inflammatory activation, and impaired protein degradation. Interconnected signaling pathways are involved in ROS-driven toxicity in neurodegenerative disorders. ROS promotes protein aggregation and neuronal damage in Alzheimer’s and Parkinson’s diseases by activating and amplifying Nrf2-related pathways through PI3K/Akt/GSK3β, p38 MAPK/NF-κB, and JNK/p53 signaling. ROS alters SOD1-linked redox balance and triggers IKK/IκB/NF-κB signaling in ALS, while autophagy and the removal of misfolded proteins are influenced by IGF1R/mTOR and PI3K/Akt-mediated Nrf2 regulation. Mutant huntingtin (mHTT) and mitochondrial malfunction raise the formation of ROS in Huntington’s disease, which damages DNA and impairs autophagy [206].

#### 8.3.1. Alzheimer’s Disease (AD)

Progressive loss of cognitive and behavioral decline is a hallmark of Alzheimer’s disease (AD), often known as dementia, which impairs routine activities. It affects 45 million individuals globally and is one of the most common neurodegenerative diseases. Although AD is typically referred to as an aging disease, it can occasionally be seen in younger people as well. Protein aggregation, intracellular tau (τ) or neurofibrillary tangles, extracellular amyloid plaques (Aβ), and the loss of synaptic connections in particular brain regions are all signs of Alzheimer’s disease [207]. The buildup of neurofibrillary tangles and amyloid β in the hippocampus causes Alzheimer’s disease. The two main categories of amyloid disease are sporadic AD (SAD) and familial AD (FAD). While *APP*, *PSEN1*, and *PSEN2* gene mutations are responsible for FAD, age, genetics, metabolism, and environmental variables all play complex roles in the pathogenesis of SAD [208]. The various theories and processes of AD are connected by oxidative stress. It is a process that happens on different paths and damages neurons. Oxidative stress is a significant contributor to AD and may perhaps be a key element in the disease’s pathophysiology [209].

Numerous investigations have demonstrated the accumulation of oxidative damage markers in proteins, lipids, and nucleic acids in postmortem AD brain tissue or cerebrospinal fluid. While ROS can interact with any biomolecule in neurons, oxidative damage to proteins in particular may play a significant role in the pathophysiology of AD by disrupting neuronal energy metabolism and proteostasis, causing aberrant redox signaling through the activation of stress-activated protein kinases (JNK, p38, and ERK 1/2), or altering redox-sensitive transcription factors through oxidative modifications. Postmortem investigations showing the translocation of NOX2 subunits p47phox and p67phox from the cytosol to the membrane have closely linked NADPH oxidase activation to the pathophysiology of AD. This activation most likely occurs in activated microglia. There is evidence linking increased ROS generation by microglial NADPH oxidase to microglial activation and the inflammatory response in the AD brain. ROS contribute to neuronal degeneration and promote microglial activation, along with other inflammatory mediators [210].

Forty elderly women with dementia and forty cognitively intact controls participated in a case–control study. A thorough geriatric evaluation that included cognitive testing, depression screening, and functional testing was performed on each participant. Blood levels of the antioxidant enzyme glutathione peroxidase, the oxidative stress marker MDA, and the antioxidant marker total antioxidant capacity were examined [211]. An analysis was done on 146 elderly adults with severe depression in a longitudinal cohort. Using an enzyme-linked immunosorbent test, biomarkers including nitrotyrosine, protein carbonyl, F2-isoprostanes, MDA, 4-hydroxynonenal, and 8-hydroxy-2′-deoxyguanosine were evaluated at baseline. Patients with plasma nitrotyrosine levels ≥ 170 nM had higher risk of AD, according to clinical applicability [212].

#### 8.3.2. Parkinson’s Disease and Oxidative Stress

Parkinson’s disease (PD) is the second most prevalent neurodegenerative disease after Alzheimer’s disease (AD) and is characterized by degeneration of dopaminergic neurons in the substantia nigra pars compacta (SNc), leading to reduced dopamine levels in the nigrostriatal pathway. The formation of Lewy bodies, composed mainly of α-synuclein and progressive neuronal loss, results in motor symptoms such as bradykinesia, rigidity, postural instability, and resting tremor [213,214,215].

Oxidative stress is considered a major contributor to PD. Patients show decreased glutathione levels and increased oxidation of lipids, proteins, and DNA in the substantia nigra, along with elevated inflammatory mediators including TNF-α and interleukins. Excess iron accumulation further enhances lipid peroxidation and neuronal injury [207]. Progressive dopaminergic (DAergic) neurodegeneration is also associated with mitochondrial dysfunction, dopamine oxidation, Ca^2+^ imbalance, neuroinflammation, and α-synuclein aggregation. Genetic mutations in *SNCA*, *PRKN*, *PINK1*, *DJ-1*, *LRRK2*, *FBXO7*, and *ATP13A2* further support the role of oxidative stress in disease development. Consequently, oxidative stress-related molecules such as DJ-1, coenzyme Q10, uric acid, 8-hydroxy-2′-deoxyguanosine, homocysteine, vitamin E, GPx, SOD, xanthine oxidase, and lipid peroxidation products have been proposed as potential PD biomarkers [216].

DNA oxidation contributes to neuronal damage and may further increase ROS production through mitochondrial dysfunction and antioxidant depletion. Altered redox homeostasis is linked to mutations in key PD-associated genes, particularly *SNCA*, where variants such as A53T, A30P, E46K, G51D, H50Q, and A53E are associated with familial forms of the disease [217]. Clinical studies measuring oxidative stress markers, including diacron-reactive oxygen metabolites (d-ROMs) and biological antioxidant potential (BAP), have demonstrated altered antioxidant capacity in PD patients, further supporting the role of oxidative imbalance in disease progression [218].

#### 8.3.3. Amyotrophic Lateral Sclerosis

Amyotrophic lateral sclerosis (ALS) or Lou Gehrig’s disease is the most common form of motor neuron disease and is characterized by progressive degeneration of upper and lower motor neurons in the cortex, brainstem, and spinal cord, ultimately leading to fatal neuromuscular impairment [219]. Approximately 90% of ALS cases are sporadic (SALS) with no clear genetic link, while 10% of familial ALS (FALS) cases are associated with inherited genetic mutations. Additionally, an enlarged GGGGCC hexanucleotide repeat in the non-coding region of the *C9Orf72* gene on chromosome 9p21 represents the most common genetic mutation linked to FALS [220].

ALS is defined by the gradual loss of lower motor neurons in the brainstem and spinal cord and upper motor neurons in the cerebral cortex. This causes weakness, stiffness, and muscle atrophy, which ultimately ends in paralysis and issues with breathing, swallowing, and speaking. Eighty percent of ALS cases begin in the limbs, while twenty percent begin in the bulbar region. ALS has no known cure, and the only medication approved to treat the condition is riluzole, which inhibits glutamate signaling. Riluzole was demonstrated to enhance limb function and reduce the progression of the disease, but it only extended patient survival by two to three months, and most patients died from respiratory failure within three to five years of diagnosis [221].

Numerous studies have reported increased oxidative damage in proteins, lipids, and DNA in postmortem neuronal tissues, as well as in plasma, urine, and cerebrospinal fluid of patients with ALS, indicating the involvement of oxidative stress mechanisms in both the central nervous system and peripheral tissues [222,223,224,225,226,227,228,229]. However, it remains unclear whether oxidative damage is a primary cause of the disease or a secondary consequence, and at which stage it appears during disease progression [230,231].

Elevated levels of protein carbonyls have been detected in the spinal cord and motor cortex of sporadic ALS patients, while increased 3-nitrotyrosine, a marker of peroxynitrite-mediated oxidative damage, has been observed in both sporadic and SOD1-linked familial ALS cases, particularly in large ventral horn neurons [224,225,232,233]. Markers of protein and lipid oxidation have also been identified in motor neurons, reactive astrocytes, and microglia/macrophages in the gray matter of sporadic ALS spinal cords but not in controls [234]. In addition, levels of 8-oxodG, a marker of oxidative DNA damage, are elevated throughout the cervical spinal cord of ALS patients, with the greatest damage observed in the ventral horn region [224,235].

Although predicting disease onset remains difficult and long-term monitoring of oxidative stress markers in patients is limited by short life expectancy, animal studies provide valuable insights. For example, activation of the Nrf2–ARE antioxidant defense system has been observed in distal muscles prior to disease onset in mutant *SOD1* (mutSOD1) mouse models. However, this model represents only a subset of cases and does not fully reflect the majority of sporadic ALS patients. Nonetheless, increased protein carbonyl formation remains a consistent indicator of oxidative damage in sporadic ALS, which accounts for most cases [222].

When compared to sporadic ALS (SALS) and normal controls (spouses of ALS patients), lymphoblast cell lines produced from FALS patients with 16 distinct mutations in the SOD1 gene show a substantial increase in intracellular ROS [236]. Similar to other discoveries from ALS brain cells/models, peripheral blood mononuclear cells (PBMCs) from ALS patients have a considerable mitochondrial dysfunction that could be used as a potent tool in ALS research [237].

### 8.4. Cardiovascular Diseases and Oxidative Stress

According to the World Health Organization (WHO), cardiovascular diseases are complex conditions that constitute the primary cause of death globally. The physiopathology of cardiovascular disorders (CVDs), which are mainly caused by atherosclerosis, includes blood vessel remodeling that may reduce blood flow, affecting the heart and the nervous system. Heart failure, hypertension, coronary artery disease, stroke, congenital heart disease, and vascular diseases are among the conditions that fall under the category of cardiovascular diseases. Obesity, diabetes, tobacco use, a sedentary and unhealthy lifestyle, and genetic predisposition are the primary risk factors for cardiovascular diseases [238].

Oxidative stress is a major contributor to the development of CVDs [239]. Endothelial cells (EC) are predominantly affected by severe oxidative stress, which causes inflammation and dysfunction of blood vessels. There is additional involvement of other blood vessel cells, including adventitia cells and vascular smooth muscle cells (VSMCs) [240]. However, endothelial dysfunction affects vasoconstriction and vasodilatation, induces EC death, increases EC adherence to monocytes, and changes EC angiogenic potential, all of which are important aspects of cardiovascular imbalance [241].

As a result, atherosclerotic lesions and plaques develop, ultimately leading to CVD [242]. Transmembrane proteins called NADPH oxidases, or NOX (for NADPH oxidase) family members, move one electron from NADPH onto molecular oxygen to produce O_2_^•−^. In contrast, NOX enzymes generate ROS as their primary biological role, whereas physiological ROS formation typically occurs as a consequence. Actually, the oxidative burst, or NOX-mediated ROS release, helps neutrophils and macrophages eliminate invasive microbes. The fact that individuals with a hereditary deficit in NOX2 suffer chronic granulomatous disease (CGD) and are unable to fight off common infections highlights the significance of ROS in the host immune response. Phagocytes had NOX2, the first NADPH oxidase. The identification of other NOX family NADPH oxidases, which are not restricted to phagocytes but are present in almost all tissues, came next [243]. Numerous lines of evidence suggest that NOX enzymes are crucial to the pathogenesis of several CVDs [244,245].

In patients with hypertension, endothelial dysfunction is associated with elevated oxidative stress, vascular inflammation, and increased ROS generation [246]. Accordingly, isolated arteries subjected to high pressure in vitro have elevated ROS levels [247], which causes endothelial damage [248]. Among the crucial factors that control blood pressure are changes brought on by oxidative stress in ECs or vascular smooth muscle cells (VSMCs) [249].

Additionally, transient elevations in blood pressure might exacerbate oxidative stress in vivo and impair endothelial function. Actually, one pathway for CVD involves inhibition of NO signaling or decreased NO bioavailability. A reduction in NO bioavailability, which results in a vasoconstrictive, pro-inflammatory, proliferative, and thrombotic state, is one sign of ROS-induced endothelial dysfunction, or EC dysfunction [247]. Vanreusel and colleagues conducted a clinical analysis that highlights the clinical relevance of oxidative stress as a biomarker in cardiovascular pathology by finding increased circulating ROS levels in people with congenital heart disease [250].

Oxidative stress plays a central mechanistic role in cardiovascular injury by promoting endothelial dysfunction, mitochondrial damage, and sustained activation of NOX enzymes. NOX-derived superoxide contributes to myocardial hypertrophy, fibrosis, and cell death through activation of MAPK signaling pathways, including ERK, JNK, and p38. In parallel, ROS reduce nitric oxide (NO) bioavailability via NOS uncoupling and peroxynitrite formation, creating a vasoconstrictive, pro-inflammatory, proliferative, and pro-thrombotic environment that accelerates atherosclerosis and vascular dysfunction [249,251].

Clinical findings further support the relevance of oxidative stress in cardiovascular pathology. Elevated circulating ROS levels have been reported in patients with congenital heart disease, suggesting their potential utility as disease biomarkers [248]. Similarly, studies in ischemic stroke patients demonstrate significantly reduced NO levels alongside increased peroxynitrite (ONOO^−^), accompanied by elevated iNOS, eNOS, and nitrotyrosine expression, indicating the involvement of oxidative and nitrosative stress in ischemic brain injury [252]. Additional clinical evidence shows higher malondialdehyde (MDA) levels and reduced total antioxidant power (TAP) in stroke patients compared with healthy controls, further supporting the contribution of oxidative imbalance to cardiovascular and cerebrovascular damage [253].

### 8.5. Lung Diseases and Oxidative Stress

Pulmonary disorders represent a major cause of morbidity and mortality, particularly in the elderly, although their prevalence is rising among younger individuals due to smoking, secondhand smoke exposure, or increasing air pollution [254]. In the lungs, oxidative stress arises from both endogenous and exogenous sources. Activated inflammatory cells such as neutrophils and macrophages generate ROS internally, while cigarette smoke, biofuel exposure, and environmental pollutants act as external contributors. The combined effect of increased oxidant burden and reduced antioxidant defenses creates a highly oxidative environment in pulmonary tissues [255].

Multiple factors, including infections, occupational exposure, air pollutants, smoking, and aging, promote lung disease through oxidative stress-mediated mechanisms [256]. Environmental exposure to fine particulate matter, respirable fibers, metal and quartz particles, ozone, and vehicle exhaust induces oxidative damage that contributes to inflammation, fibrosis, and carcinogenesis [257]. Cigarette smoke further intensifies oxidative injury by damaging mitochondrial function in bronchial epithelial cells, enhancing MMP-9 activity linked to emphysema, and activating EGFR signaling pathways that impair lung function and promote airway hypersecretion [258].

In chronic obstructive pulmonary disease (COPD), oxidative stress is a central pathogenic driver. It promotes chronic inflammation, cellular senescence, defective autophagy, reduced DNA repair, and steroid resistance. These effects are mediated through activation of p38 MAPK and NF-κB signaling, decreased sirtuin-1 and HDAC2 expression, increased TGF-β release leading to airway fibrosis, reduced antiprotease activity causing emphysema, and enhanced mucin gene expression resulting in mucus hypersecretion [255]. Collectively, these mechanisms accelerate disease progression, increase exacerbations, and contribute to systemic complications.

Oxidative stress also plays a critical role in infectious lung diseases such as pneumonia. Hyperglycemia-associated oxidative stress, altered redox signaling, and inflammatory responses contribute to disease onset and progression. Increased mitochondrial O_2_^•−^ production, protein glycation, and activation of redox-sensitive pathways promote structural and functional lung alterations. Excess mitochondrial ROS can induce apoptosis of alveolar epithelial cells, damaging the alveolar membrane. Subsequent fibroblast activation and excessive extracellular matrix deposition may lead to structural remodeling and impaired lung function [259].

In asthma, oxidative stress intensifies airway inflammation by activating redox-sensitive signaling pathways and increasing the release of inflammatory mediators. Elevated ROS levels correlate with higher inflammatory cell counts and disease severity [257]. Studies have shown that antigen exposure in asthmatic patients increases eosinophil counts and O_2_^•−^ production, identifying eosinophils as a major source of ROS involved in allergic airway injury [260]. Increased production of ROS and reactive nitrogen species by inflammatory cells has been consistently observed in asthmatic airways. Notably, human thioredoxin-1 (TRX1) has demonstrated protective effects in experimental models by reducing airway hyperresponsiveness, limiting inflammatory cell infiltration, and attenuating airway remodeling [261].

Inflammatory cells involved in bronchial asthma also produce ROS, including hydrogen peroxide, which contributes to disease severity. Serum levels of reactive oxygen metabolites (ROM) have been shown to correlate significantly with the degree of airway obstruction, white blood cell and neutrophil counts, IL-6 levels, and severe exacerbations [262]. Similarly, patients with idiopathic pulmonary fibrosis (IPF) exhibit significantly elevated serum oxidative stress levels compared to controls [263].

At the molecular level, ROS activate key signaling pathways such as MAPK (ERK, JNK, and p38), PI3K/Akt, PKC, and phospholipase A_2_, leading to enhanced lung injury. These pathways stimulate redox-sensitive transcription factors, including NF-κB, HIF-1α, and AP-1, which promote the expression of cytokines, chemokines, and adhesion molecules. ROS can also influence histone acetylation, further enhancing the transcription of inflammatory genes. Together, these processes contribute to airway inflammation, epithelial damage, mucus hypersecretion, and smooth muscle dysfunction in lung diseases such as asthma, COPD, and acute lung injury [264].

### 8.6. Liver Pathogenesis and Oxidative Stress

ROS, produced during metabolic processes and the biotransformation of xenobiotics, are especially harmful to the liver. Oxidative stress alters inflammatory pathways, impacts liver function, and exacerbates diseases. Acute liver injury and the pathophysiology of common infectious or metabolic chronic liver disorders, including viral hepatitis B or C, alcoholic fatty liver disease, non-alcoholic fatty liver disease (NAFLD), and non-alcoholic steatohepatitis (NASH), are therefore linked to oxidative stress. Furthermore, the development of liver disease into cirrhosis, hepatocellular carcinoma (HCC), and hepatic fibrosis is significantly influenced by oxidative stress [265].

Fat buildup, organelle stress and hepatocyte mortality, immune cell infiltration and activation, and fibrogenesis triggered by hepatic stellate cells are some of the mechanisms involved in the pathophysiology of alcoholic liver disease (ALD). Oxidative stress is said to promote and/or stimulate these processes. The metabolism of ethanol to acetaldehyde and acetate, along with associated activities that convert NAD^+^/NADP^+^ to NADH/NADPH, can generate ROS. ROS generated by these mechanisms can directly cause hepatocyte damage or promote fat storage. Innate immune cells like neutrophils and macrophages are activated and drawn in by the cytokines, chemokines, and DAMPs released by injured hepatocytes. Via NADPH oxidase, activated neutrophils and macrophages can also generate ROS. Acetaldehyde and ROS can generate protein and DNA adducts that can promote inflammation, carcinogenesis, and liver damage [266].

NAFLD is a complex and multifactorial disease that ranges from simple steatosis to non-alcoholic steatohepatitis (NASH), which can progress to cirrhosis and hepatocellular carcinoma (HCC). It is associated with genetic, epigenetic, and environmental factors, with insulin resistance (IR) considered a central initiating event. IR promotes hepatic lipid accumulation through increased lipogenesis and reduced free fatty acid oxidation, making the liver more susceptible to inflammation and hepatocellular injury. This subsequent pathogenic stimulus, largely driven by oxidative stress, contributes to the transition from steatosis to NASH and fibrosis [267].

The majority of NAFLD patients have been found to have impaired redox status in multiple investigations, as evidenced by elevated levels of lipid peroxidation products and oxidative stress biomarkers in blood and plasma. Accordingly, several oxidative stress markers, including 8-isoprostane, 8-oxodG, and TBARS/MDA, have been examined in serum/plasma and liver samples from individuals with non-alcoholic fatty liver disease (NAFLD), and elevated levels or activity for the majority of these markers have been documented. MDA and 4-hydroxynonenal (4-HNE), which are frequently used as indicators of lipid peroxidation in clinical practice, are two additional peroxidized lipids elevated in NAFLD patients [268]. Furthermore, a recent study indicates that decreased plasma free thiol levels may serve as a biomarker for non-alcoholic fatty liver disease (NAFLD) and be a worldwide indicator of the systemic load of reactive species [269].

While the majority of research indicates that NAFLD patients have lower levels of hepatic antioxidant enzymes [270], some studies have found that NAFLD patients have both higher and lower blood levels of antioxidant enzymes such as SOD, GPx, and GSH [271,272]. The pathophysiology of NAFLD, which initially involves an adaptive antioxidant response to increased ROS generation, followed by depletion of the antioxidant system and decreased antioxidant enzyme levels, may help explain these contradictory findings [273].

Hepatocyte apoptosis and necrosis are caused by excess ROS. ROS exacerbate liver inflammation by activating stress-responsive signaling pathways such as NF-κB, p38 MAPK, and JNK, which promote the generation of pro-inflammatory cytokines. Furthermore, through TGF-β and redox-sensitive signaling, oxidative stress stimulates the activation of hepatic stellate cells, a crucial step in fibrogenesis. This leads to the deposition of extracellular matrix and the development of cirrhosis and fibrosis. In diseases including NAFLD, alcoholic liver disease, and viral hepatitis, a vicious loop that propels disease development is created when mitochondrial dysfunction further maintains ROS production [265].

### 8.7. Erectile Dysfunction and Oxidative Stress

Erectile dysfunction (ED) is defined as the persistent inability to achieve or maintain a penile erection sufficient for satisfactory sexual performance. During sexual stimulation, non-adrenergic, noncholinergic (NANC) nerve fibers release nitric oxide (NO^•^), which stimulates the production of 3′,5′-cyclic guanosine monophosphate (cGMP). This activates protein kinase pathways, reduces intracellular Ca^2+^ levels, and promotes smooth muscle relaxation. As a result, blood fills the lacunar spaces of the corpora cavernosa, compressing subtunical venules and restricting venous outflow (veno-occlusion). The erection subsides when cGMP is degraded by phosphodiesterase type 5 (PDE5). Disruption at any stage of this signaling cascade can impair erectile function [274].

Oxidative stress plays a significant role in ED, particularly under diabetic conditions, where increased ROS production has been strongly associated with vascular dysfunction [275]. Clinical studies indicate that combining sildenafil with the antioxidant L-carnitine reduces markers of endothelial dysfunction and monocyte oxidative activity in diabetic patients with ED [276]. Similarly, vitamin E has been shown to enhance the therapeutic efficacy of PDE5 inhibitors, supporting the role of ROS scavenging in preserving erectile function [277].

Mechanistically, oxidative stress contributes to ED through lipid peroxidation, protein and DNA oxidation, impaired NO^•^ synthesis, and reduced NO^•^ bioavailability. Chronic inflammation and hypoxia further strengthen the link between endothelial dysfunction and erectile dysfunction. Persistent inflammation damages endothelial capacity to regulate vascular responses necessary for erection, while reduced erectile activity lowers oxygen tension in penile tissues, intensifying pro-inflammatory and oxidative processes [278].

Excess ROS disrupt important vascular and endothelial signaling pathways involved in ED. Excess ROS generated from NADPH oxidase and mitochondria reduce NO^•^ bioavailability through eNOS uncoupling and peroxynitrite formation, thereby impairing the NO–cGMP signaling pathway essential for smooth muscle relaxation and penile erection. In addition, ROS activate NF-κB, MAPK, and JNK pathways, leading to endothelial injury, inflammation, and cavernosal cell apoptosis. Oxidative stress also promotes vascular remodeling and TGF-β-mediated fibrosis, which further lowers penile blood flow [278,279,280].

### 8.8. AIDS and Oxidative Stress

HIV-1 causes immunodeficiency by infecting and killing key immune cells, such as dendritic cells, T-helper cells, and macrophages, through various mechanisms. In those with acquired immunodeficiency syndrome (AIDS), HIV-1 infection weakens the immune system over time, allowing cancer and other potentially fatal opportunistic infections to proliferate. HIV uses the enzymes reverse transcriptase and integrase, which are encoded by the virus, along with cellular cofactors, to reverse-transcribe the RNA genome into double-stranded DNA, transport it into the cell nucleus, and integrate it into the chromosomes once it has entered the target cell. The virus may become dormant after integration, enabling the infected cells to evade immune system recognition [281]. In HIV infected people, there has been evidence of immunological overactivation, inflammation, DNA damage, genomic instability, premature CD4^+^ T-cell aging, oxidative stress, and failed memory CD4^+^ T-cell responses. Additionally, it has been demonstrated that people with HIV produce excessive amounts of ROS during oxidative phosphorylation, which results in cancer, neurological disorders and CVD, accelerated aging, and damage to mitochondrial DNA [282].

People with HIV have significantly higher levels of oxidized nucleic bases, such as 8-oxoG, and lipid peroxidation products, such as MDA, in plasma, and alkanes in breath output, as well as increased ROS generation in monocytes [281,282,283]. HIV-positive patients exhibit lower levels of antioxidant enzymes, while ART-naïve people have higher amounts of MDA and lower levels of blood SOD, GPx, and CAT. Increased antioxidant enzyme activity reduces lipid peroxidation, raises CD4^+^ counts in ART-naïve patients, and enhances HIV-positive health. Viral replication, oxidative stress, and increased oxidized glutathione (GSSG) levels are caused by low glutathione reductase (GR) activity [282]. The HIV-infected cell cultures also showed a considerable increase in ROS levels. Subsets of CD4^+^ and CD8^+^ T-lymphocytes showed the greatest reduction in overall antioxidant capacity, with lower CD4^+^ T-cell numbers being associated with more severe oxidative stress [281].

Cytochrome P2A6 (CYP) also causes increased oxidative stress and viral load in smokers, HIV+ only, and HIV-positive smokers compared to HIV-negative non-smokers. According to recent research, smoking-mediated HIV-1 pathogenesis, including HIV-1 replication and drug–drug interactions, may include a novel route called CYP-mediated oxidative stress. Therefore, CYP and CYP-related oxidative stress pathways could be viable targets for the development of new medications for smokers with HIV-1 [284]. Compared to ART-experienced and control groups, ART-naïve patients exhibited lower superoxidase dismutase (SOD) activity, and manganese showed a strong negative connection with SOD activity and a positive correlation with CD4^+^ count [281].

### 8.9. Kidney Diseases and Oxidative Stress

Over 2 million people worldwide suffer from chronic kidney disease (CKD), and the majority of them get hemodialysis (HD) or other types of renal replacement treatment. CV disease is the primary cause of death for persons with CKD. Diabetes, hypertension, and dyslipidemia are well-known risk factors that are closely linked to CV disease in individuals with chronic kidney disease [285]. A permanent change in the kidney’s structure or function causes CKD. Kidney disease is defined as pathologic abnormalities seen by imaging studies or renal biopsies, abnormalities in urine sediment, or high urinary albumin excretion rates. Additional health consequences of CKD include anemia, or low red blood cell count, increased infection rates, elevated blood levels of potassium, phosphorus, and Ca^2+^, decreased appetite or consumption, depression, or a lower quality of life. Renal failure, the final stage of CKD, is potentially lethal if kidney replacement therapy is not employed to treat it. A diagnosis of CKD is made in an adult patient with a glomerular filtration rate (GFR) of less than 60 mL/min/1.73 m^2^ for 3 months or more and evidence of renal structural damage [286,287].

CKD patients have higher levels of circulating biomarkers, including proteins, lipids, and nucleic acids. The antioxidant defense is compromised because CKD patients are unable to eliminate ROS, and atherosclerotic CKD patients have oxidative stress markers. It is frequently debated how oxidative stress contributes to the development of cardiovascular problems in uremic individuals. Increases in circulating oxidative stress biomarkers have been found to indicate that a pro-oxidant state can develop as early as CKD stage 3. Accordingly, oxidative stress can also lead to a deterioration in renal function through fibrosis, glomerular filtration barrier degradation, hypertension, and inflammation (NF-kB activation) [285].

Uremic toxins significantly increase oxidative stress in CKD. ROS, NO^•^, and oxidative stress markers like MDA, peroxynitrite (ONOO^−^), and advanced glycation end products (AGEs) are all on the rise. These substances interact with AGE receptors to activate the nuclear factor kappa-light-chain-enhancer of activated B cells (NF-κB), which, in turn, causes an increase in cytokines and adhesion molecules. Reduced activity of the transcription factor nuclear factor erythroid 2-related factor 2 (Nrf2) and, as a result, decreased expression of cytoprotective and antioxidant enzymes like SOD, CAT, and (NQO1) are indicative of the oxidative stress condition [288].

ROS have been implicated experimentally as the main mediators in the pathophysiology of kidney injury brought on by ischemia, toxins, and antibody–antigen interactions at the glomerular and tubular levels. Additionally, subclinical inflammation induced by oxidative stress causes endothelial dysfunction and vascular aging, thereby reducing NO^•^ availability. Oxidative stress levels are much higher in CKD patients than in healthy people, and these alterations worsen as eGFR falls, demonstrating clear variations across CKD stages. Furthermore, the three molecular lines studied were equally affected by variations in oxidative stress parameters. While antioxidant capacity declines, the process starts in the early stages of renal disease and intensifies as the condition worsens [289].

ROS cause renal fibrosis by activating important signaling cascades such as NF-κB, MAPK, and TGF-β/Smad pathways, which, in turn, promote the generation of pro-inflammatory cytokines, mesangial cell proliferation, and extracellular matrix buildup. Oxidative stress is further increased when ROS inhibit the Nrf2 antioxidant defense system. In addition to causing podocyte death, endothelial dysfunction, and tubular epithelial-to-mesenchymal transition, and persistent oxidative damage also contributes to glomerulosclerosis and the steady decline in kidney function that characterizes chronic kidney disease [290,291,292].

### 8.10. Cystic Fibrosis and Oxidative Stress

Cystic fibrosis (CF) is the most common fatal autosomal recessive genetic disorder in the Caucasian community, with an incidence of approximately one case per 2500 live births. It is caused by mutations in the *cystic fibrosis transmembrane conductance regulator* (*CFTR*) gene, leading to malfunction of the CFTR protein. This defect disrupts chloride ion transfer and alters mucus hydration in organs, including lung, pancreas, and other organs, resulting in thick, viscous secretion [293]. In the lungs, impaired mucociliary clearance promotes microbial colonization, recurrent infections, and persistent inflammation, creating a vicious cycle that progressively damages airway epithelial cells [294].

Oxidative stress plays a central role in CF-associated lung damage. Elevated oxidant levels, together with reduced antioxidant defenses, contribute to chronic cellular damage and impaired airway remodeling. This redox imbalance arises from both genetic factors, including CFTR dysfunction, and acquired factors such as repeated infections and sustained inflammatory responses [295]. Evidence indicates that CF patients exhibit increased oxidative stress markers despite adequate dietary antioxidant intake. In a comparative study measuring plasma 8-iso-PGF2α, antioxidant vitamins (E, C, and β-carotene), and erythrocyte antioxidant enzyme activities (GPx and SOD), CF patients showed significantly higher oxidative stress levels than healthy controls, suggesting that immune-driven inflammation is a major contributor to oxidative damage in CF [296].

### 8.11. Urinary Tract Infection and Oxidative Stress

Clinical manifestations of urinary tract infections (UTIs) range from asymptomatic bacteriuria to urosepsis. UTIs are an issue for people of all ages, from newborns to adults and senior citizens. Antioxidant enzymes like CAT and SOD are included in qualitative tests that are currently used to diagnose UTIs [297]. The most prevalent bacterial infection during pregnancy is a urinary tract infection (UTI), which can lead to major obstetric problems. The most frequent reason for admission to obstetrical wards is UTI, which has been reported to affect 20% of pregnant women. One in three women who are of childbearing age is predicted to get a UTI. Infectious organisms in the genitourinary tract that cannot be attributed to contamination are what define UTIs. The second most frequent pregnancy-related medical problem is a UTI [298].

The two types of UTIs are uncomplicated and complicated bladder infections. Uncomplicated cystitis, another name for UTIs, is a disease that only affects the lower urinary tract. The majority of those with uncomplicated cystitis are young. However, complicated UTI is a general term that includes a number of illnesses, such as catheter-associated UTI, pyelonephritis, and complicated cystitis. Pyelonephritis is caused by inflammation of the renal parenchyma as a result of the infection migrating towards the kidneys. Systemic injuries and damages can result from persistent inflammation that causes tubular damage, which can cause interstitial edema, nephritis, and acute kidney injury. The most common causes of complicated disease are pathology, underlying renal catheterization, compromising circumstances, and elderly persons [299].

In contrast to patients with negative urine cultures, individuals with positive cultures displayed significantly higher serum MDA levels and lower glutathione reductase and SOD activity, suggesting oxidative stress brought on by UTI [300]. Between the first and third trimesters of pregnancy without a UTI, there was an increase in CAT, SOD activity, and LPO levels. However, during pregnancy with UTI, LPO levels rose from the first to the third trimester, whereas CAT and SOD activity decreased (*p* < 0.01). Oxidative stress is brought on by pregnancy, and a urinary tract infection during pregnancy might make it worse [301].

### 8.12. Aging and Oxidative Stress

The steady loss of organ and tissue function is a hallmark of aging. The foundation of the oxidative stress theory of aging is the idea that the accumulation of RONS-induced damage causes age-related functional deficits. Oxidative stress is also linked to sarcopenia and frailty, as well as a number of age-related diseases, including cancer, neurological disorders, chronic renal disease, cardiovascular diseases, and chronic obstructive pulmonary disease. The accumulation of different cellular and molecular degenerations during aging is a progressive and complex physiological process that results in worsened biological events and a slow reduction in resistance and adaptation to metabolic stress. The body’s capacity to function at its best, both physically and mentally, gradually deteriorates with age [302].

The aging mechanism is predicated on the idea that high intracellular free radical concentrations alter cellular structure and function, disrupt regeneration, and cause mitochondrial malfunction. The main contributors to the overproduction of cellular oxidative stress are the mitochondria and the NOX system. NOX expression and/or activity were elevated in chronic degenerative disorders. Cellular senescence, which is defined by the termination of cellular growth in both normal and pathological processes, has been hypothesized to result from increased RONS levels and oxidative stress [303,304,305].

Furthermore, a variety of triggers, including the release of degenerative matrix metalloproteases (MMPs), insoluble extracellular matrix (ECM) components, and soluble chemokines, cytokines, and growth factors, can cause senescence-associated secretory phenotype (SASP) [306].

Cellular senescence results from chronic ROS overproduction and speeds up telomere shortening. At the molecular level, ROS promote inflammation, apoptosis, and poor tissue regeneration by activating pathways like p53/p21, NF-κB, and MAPK. In addition to suppressing antioxidant defenses, oxidative stress also modifies autophagy and proteostasis, which results in the buildup of damaged macromolecules. These mechanisms eventually lead to metabolic inefficiency, chronic inflammation, stem cell exhaustion, and a steady deterioration in organ function, all of which are factors in biological aging and age-related diseases [1].

### 8.13. COVID-19 and Oxidative Stress

A potentially fatal virus, coronavirus disease-2019 (COVID-19) is brought on by the severe acute respiratory syndrome coronavirus 2 (SARS-CoV-2). Their involvement in pneumonia has been better understood and detected thanks to developments in molecular diagnostic tools. The positive-sense, single-stranded RNA virus known as SARS-CoV-2, which was discovered during the 2019 Wuhan outbreak, is a member of the Betacoronavirus genus and has a genomic size of roughly 29.8 kb. According to phylogenetic analysis, it is closely related to coronaviruses that resemble SARS and most likely came from animals, especially bats, before spreading to people [307,308].

The pathophysiology of COVID-19 is significantly influenced by oxidative stress, which can also be linked to the aftereffects of a severe SARS-CoV-2 infection. Inflammation biomarkers and OS were assessed in blood samples from patients with post-acute COVID-19 sequelae (PASC). IL-6 and IL-8 levels were elevated in PASC patients. Both COVID-19 groups showed lower levels of CAT and SOD. Inflammatory indicators and OS characteristics were shown to be correlated. PASC patients have substantial oxidative stress, which may make disease consequences worse [309]. A study sought to evaluate oxidative stress metrics and antioxidant status as possible key factors in individuals with severe, non-severe, and asymptomatic COVID-19. Compared with the severe and normal groups, non-severe patients had significantly lower levels of FRAP, G6PD activity, and SOD activity. MDA content and NO metabolite levels were observed to be significantly higher in severe patients than in the non-severe group. It appears that COVID-19 patients had an imbalance between oxidants and antioxidants, favoring oxidant markers [310].

ROS had a significant impact on both the inflammatory response and viral replication. Numerous inflammatory and cell-death pathways are centrally regulated by excessive ROS produced during SARS-CoV-2 infection. It produces ferroptosis by iron-driven lipid peroxidation and GPX4 inhibition, enhances NETosis by facilitating NE and MPO-mediated chromatin decondensation, and initiates apoptosis by inhibiting the PI3K/AKT/mTOR pathway and activating caspases. Additionally, ROS promotes autophagy and mitophagy and activates the NLRP3 inflammasome to induce pyroptosis, all of which increase tissue damage, inflammation, and immunological dysregulation [311].

## 9. Integrative Redox Perspectives and Emerging Translational Directions

Oxidative stress reflects a disruption of tightly regulated redox networks rather than simply excessive accumulation of reactive oxygen species (ROS). Accumulating evidence demonstrates that ROS function as spatially and temporally controlled signaling mediators, with biological outcomes determined by concentration, localization, and duration of exposure [19]. ROS are key regulators of cell fate by orchestrating multiple regulated cell death (RCD) pathways. Their effects depend on concentration, subcellular localization, and exposure duration. They can initiate or modulate diverse death programs and can facilitate crosstalk between them and determine inflammatory and non-inflammatory outcomes. Rather than being a mere byproduct of cellular stress, redox imbalance emerges as an integrative mechanism that links diverse RCD pathways and influences disease progression [312].

The biological significance of efficient redox regulation is highlighted by comparative studies carried out in scallop species exposed to ionizing radiation. Longer-lived species exhibited stronger antioxidant and autophagy responses, accompanied by lower ROS accumulation, whereas shorter-lived species showed impaired stress-response pathways, higher oxidative burden, and reduced survival. These observations suggest that adaptive redox buffering capacity is a key determinant of resilience, longevity, and stress tolerance across biological systems [313].

From a translational perspective, this mechanistic understanding has driven the development of redox-engineered nanotherapeutics designed to selectively modulate ROS levels in pathological conditions. Recent nanomaterials can specifically amplify ROS within tumor cells, disrupting redox balance and inducing oxidative damage, often in combination with chemodynamic (CDT), photodynamic (PDT), or sonodynamic (SDT) therapies [314]. For example, Bi@MOF-801 enables self-driven ROS generation through spontaneous electron transfer without the need for external stimulation, while simultaneously depleting intracellular GSH and enhancing apoptotic responses [315]. Similarly, nMOF-based platforms combined with metal or metal oxide/peroxide nanoparticles can alleviate tumor hypoxia by catalyzing H_2_O_2_ decomposition or supplying oxygen, reflecting a shift toward microenvironment-responsive redox engineering for improved therapeutic efficacy [316].

Beyond chemical and metabolic modulation, emerging technologies demonstrate that ROS production can be externally programmed. Peizodynamic systems, for example, provide mechanical control over ROS production while integrating scavenging functions to preserve redox balance, establishing a dynamic redox-balancing paradigm that aligns with physiological homeostasis [317].

## 10. Conclusions and Future Directions

Oxidative stress is a key mechanism that contributes to the development and onset of many human diseases. Reactive oxygen species (ROS) are essential for physiological signaling and immune defense; however, their uncontrolled overproduction can lead to cumulative molecular damage and harmful outcomes. Redox regulation is a complex process that involves dynamic networks of ROS, enzymes, organelles, and the microenvironment, all of which influence metabolism, immunity, and overall health. Recent studies and advanced imaging technologies allow for precise spatiotemporal mapping of ROS-dependent pathways, accelerating the search for biomarkers and therapeutic targets. A more integrated understanding of redox biology, along with improved detection tools and targeted therapeutic strategies, will be crucial for enhancing the diagnosis, treatment, and prevention of diseases driven by oxidative stress. This review summarizes the current understanding of ROS biology and highlights the interplay between endogenous metabolic pathways, environmental exposures, and redox-sensitive signaling networks. Despite significant advances in knowledge of the mechanisms underlying oxidative damage, there are still considerable challenges in translating these findings into practical treatment strategies. For greater clinical success, promising treatments should prioritize selective redox pathway modulators, targeted antioxidants, and enhanced delivery methods. However, the lack of sensitive, standardized ROS detection techniques and inadequate mechanistic clarity restrict translation. Focused biomarker identification, pathway-specific study, and the creation of clinically useful redox-based treatments are necessary for future advancement.

## Figures and Tables

**Table 1:** Classification of reactive oxygen and nitrogen species (ROS/RNS).

Type	Species	Chemical Formula	Primary Source	Biological Role	Reference
Radical	Superoxide	O_2_^•−^	Mitochondrial electron transport chain leakage; NADPH oxidases (NOX); activated immune cells	Signaling molecule, regulating numerous biological processes including apoptosis, aging, and senescence, associated with the development of several diseases	[30,31,32]
Radical	Hydroxyl radical	^•^OH	Multiple reactions Including Haber–Weiss reaction and Fenton reaction	Can reduce disulfide bonds in proteins, specifically fibrinogen, resulting in their unfolding and scrambled refolding into abnormal spatial configurations in pathogenesis of diseases	[33,34]
Non-radical	Hydrogen peroxide	H_2_O_2_	Generation by major sources, the NADPH oxidases or complex III of the mitochondrial respiratory chain	A second messenger in insulin signaling and in several growth factor-induced signaling cascades, proliferation, differentiation, tissue repair, inflammation, circadian rhythm, and aging	[35,36]
Radical	Peroxyl radical	ROO^•^	Formed by radical interactions with two biological constituents: lipids and nucleobases	To oxidize cellular constituents to intermediates that may play a role in toxicity and carcinogenicity	[1,37]
Non-radical	Hypo Chlorus acid radical	HOCl	The main reaction catalyzed by myeloperoxidase under physiological conditions, is the oxidation of the Cl^−^ anion by H_2_O_2_ to give hypochlorous acid	in numerous pathologies, in the chlorination of tyrosine residues, antimicrobial function	[38]
Non-radical	Ozone	O_3_	Singlet oxygen (^1^O_2_)–driven oxidant formation by antibodies or amino acids, and by neutrophils during bacterial killing	Interactions with lung tissue, oxidation or peroxidation of biomolecules, and a beneficial effect on models of liver injury	[39,40]

**Table 2:** Sources of reactive oxygen species (ROS): Endogenous and exogenous origins.

Source Type	ROS Source	Site	Types of ROS Generated	Mechanistic Relevance	Reference
Exogenous—Physical agents	Ultraviolet (UV) radiation	Skin and ocular tissues	O_2_^•−^, ^1^O_2_, ^•^OH, and H_2_O_2_	Oxidative damage of lipids, proteins, DNA, ECM; triggers redox-sensitive signaling (MAPK, NF-κB) → inflammation, photoaging, carcinogenesis, ocular damage	[61]
Exogenous—Ionizing radiation	X-rays, γ-rays	Skin, bone marrow, gastrointestinal tract, lungs, brain, reproductive organs	O_2_^•−^, ^1^O_2_, ^•^OH, and H_2_O_2_,	Radiolysis of water generates reactive species that damage DNA, lipids, proteins; triggers mitochondrial dysfunction, persistent oxidative stress, bystander effects and long-term genomic instability.	[62,63]
Exogenous—Environmental/Pollution/External Oxidants	Air pollutants, smoke, chemical oxidants, environmental toxins	Airway epithelium, lung tissue, immune cells in lung	O_2_^•−^, ^•^OH, H_2_O_2_	Exogenous oxidants induce airway oxidative stress, driving inflammation, tissue damage, and disease progression in asthma and COPD.	[64]
Exogenous—Environmental/Smoke and Pollutants	Cigarette smoke (active/passive)	Lung/airway epithelium, alveolar tissue, vascular endothelium of lungs	High ROS load (free radicals present in smoke) + induced ROS (O_2_^•−^, peroxides, radicals)	Persistent oxidative stress damaging lipids, proteins, DNA; triggers inflammation—contributing to COPD, lung cancer, fibrosis and impaired respiratory health	[65]
Exogenous—Environmental/Heavy-metal exposure	Toxic metals (Cd, Pb, Hg, As, Cr, etc.)	Liver, kidney, brain, systemic tissues	O_2_^•−^, ^•^OH, H_2_O_2_	Toxic metals induce oxidative stress by depleting glutathione, inhibiting antioxidant enzymes, and promoting ROS generation via redox cycling and mitochondrial dysfunction.	[66,67]
Exogenous—Occupational particulate exposure	Crystalline silica (silica dust)	Lung (alveolar macrophages, epithelial cells)	O_2_^•−^, ^•^OH, H_2_O_2_	Silica activates macrophage-derived ROS and impairs antioxidant defenses, driving inflammation, fibrosis, and lung injury.	[68]
Exogenous—Drugs and xenobiotics	Drug metabolism (e.g., chemotherapeutics, xenobiotic drugs)	Liver, kidney, heart (and other metabolizing/target organs)	O_2_^•−^, H_2_O_2_ (and related ROS)	CYP-mediated xenobiotic metabolism “leaks” electrons → ROS overproduction, antioxidant depletion, biomolecular damage and organ toxicity	[69]
Endogenous—Metabolic	Mitochondrial electron transport chain (ETC)/Oxidative phosphorylation	Inner mitochondrial membrane/matrix	O_2_^•−^, H_2_O_2_	Electron leakage during ATP production produces ROS—links metabolism to redox signaling; dysregulation → oxidative stress and cell damage.	[70]
Endogenous—Metabolic	NADPH oxidase (NOX family)	Plasma membrane/endosomes/Endoplasmic Reticulum (varies with isoform)	O_2_^•−^, H_2_O_2_	Dedicated electron-transfer from NADPH to oxygen produces ROS for signaling or defense; dysregulation leads to oxidative stress and tissue damage	[71]
Endogenous—Metabolic/organelle-based	Peroxisome (fatty acid β-oxidation and other oxidases)	Peroxisomal compartment	H_2_O_2_ (from acyl-CoA oxidases), other ROS/RNS (from oxidases, lipid metabolism)	Peroxisomal oxygen-consuming metabolism generates ROS; peroxisomal antioxidant systems regulate redox balance; imbalance contributes to oxidative stress	[72,73]
Endogenous—Immune enzymatic	Myeloperoxidase (MPO) from activated neutrophils	Neutrophil granules → phagosomes/extracellular space during inflammation	HOCl, halogen radicals, secondary ROS/RNS	MPO-catalyzed halogenation oxidizes host biomolecules causing tissue damage and inflammation	[74]
Endogenous—Nitrosative	Uncoupled eNOS/iNOS	Cytosol, plasma membrane	O_2_^•−^, ONOO^−^	NO–ROS imbalance → nitrosative stress, protein nitration, mitochondrial dysfunction, neuronal damage	[75]

**Table 3:** Major ROS-responsive signaling pathways and their cellular outcomes.

Signaling Pathway	ROS Involved	Cellular Outcome	Key Molecular Targets	Disease Relevance	Reference
p38 MAPK pathway	H_2_O_2_, O_2_^•−^	Oxidative stress, mitochondrial dysfunction, apoptosis	p38 MAP kinase	Ischemia/reperfusion injury, cardiac tissue damage	[118]
p38 MAPK (ROS-activated)	H_2_O_2_	Cell cycle arrest, growth inhibition	p38 MAPK, H-Ras	Suppression of oncogenic H-Ras–driven malignant transformation	[119]
NF-κB	H_2_O_2_, O_2_^•−^	Transcription of inflammatory, survival, and antioxidant genes (SOD, GPx)	IKK complex (IKKβ), IκBα, NF-κB (p50, p65, p52, RelB, c-Rel)	Cancer, inflammatory diseases, neurodegeneration, arthritis	[120,121,122]
NF-κB	H_2_O_2_	Modulation of NF-κB activation, nuclear translocation and transcription of inflammatory, survival, and antioxidant genes	IKK complex (IKKβ), IκBα, NF-κB subunits (p65/p50), redox-sensitive cysteine residues	Chronic inflammation, cancer, autoimmune diseases, neurodegeneration	[123]
PKC activation	H_2_O_2_ and other ROS	Oxidative modification releases PKC autoinhibition, kinase activation independent of DAG/Ca^2+^	Cysteine-rich zinc-finger (C1) domain of PKC regulatory region	Aberrant PKC activation contributing to tumor promotion under oxidative stress	[124]
Glucose-PKC signaling in vascular smooth muscle cells	ROS	Up-regulation of vascular permeability factor (VEGF) expression and secretion	PKC isoforms (glucose-activated), vascular permeability factor/VEGF mRNA and peptide	Diabetic vasculopathy, increased vascular permeability in diabetes	[125]
TNF family	ROS (H_2_O_2_ as signaling mediator)	AP-1 activation, transcription of inflammatory, stress-response, and apoptotic genes	TNF receptors, TRAF proteins, ASK1, MKK4/7, JNK, MKK3/6, p38, c-Jun/c-Fos (AP-1)	Inflammation, immune regulation, cancer, apoptosis	[126]
TNF family	ROS generated via mitochondrial pathways and NADPH oxidases	Sustained activation of JNK and other MAPKs, induction of apoptosis or necrosis	ROS modulate redox-sensitive signaling components, e.g., inactivation of phosphatases that deactivate JNK, alteration of redox state of signaling proteins	Inflammation, tissue injury, cytotoxicity in response to TNFα, implicated in chronic inflammatory diseases, degenerative conditions	[127]
EGFR/PI3K/Akt pathway	ROS	Pro-survival, inflammatory signaling	EGFR (activated), PI3K, Akt (activated)	Lung epithelial inflammation	[128]
EGFR/PI3K/Akt pathway	Increased intracellular ROS (mitochondrial-derived)	Inhibition of pro-survival signaling, mitochondrial dysfunction, GSH depletion, caspase-dependent apoptosis	EGFR (phosphorylation), PI3K, Akt (p-Akt), mTOR (p-mTOR)	Overcoming drug resistance in EGFR-mutant, erlotinib-resistant non-small cell lung cancer (NSCLC)	[129]
JNK1/2	ROS	COX-2/PGE_2_ induction of inflammation	JNK1/2, AP-1, FoxO1	Lung injury	[128]
ROS-dependent JNK/MAPK pathway activated by 2′-Hydroxycinnamaldehyde	Increased intracellular ROS (mainly H_2_O_2_)	JNK activation → mitochondrial dysfunction, caspase-dependent apoptosis in HL-60 leukemia cells	JNK, c-Jun, MAPK pathway components; mitochondrial apoptotic regulators (Bax↑, Bcl-2↓); caspases	Potential therapeutic approach for acute promyelocytic leukemia via selective ROS-mediated apoptosis	[130]
Ca^2+^	H_2_O_2_, O_2_^•−^	Ca^2+^ overload increased mitochondrial ROS, mitochondrial dysfunction	Mitochondrial electron transport chain (ETC), mitochondrial Ca^2+^ uniporter (MCU), dehydrogenases, ATP synthase, ANT, etc., NOX2, NOX5	Aging-associated cardiomyopathy, neurodegeneration, Vascular aging, hypertension, atherosclerosis	[131]

**Table 4:** Approaches used for ROS detection in clinical and preclinical studies.

Approach	Ligands	Target ROS	Application	Limitation	Reference
Spin-trapping	DMPO, DEPMPO	^•^OH, O_2_^•−^	EPR-based direct radical detection in tissues, cells, and biological fluids	Spin adducts may degrade to EPR-silent products; limited in vivo stability; requires specialized equipment	[162,163]
Fluorescence	DCFH-DA, DHE, Amplex Red, CellROX	General ROS (DCFH-DA), O_2_^•−^ (DHE), H_2_O_2_ (Amplex Red)	Live-cell fluorescence imaging, microscopy, flow cytometry	Auto-oxidation, nonspecific reactivity, probe loading variability; environmental sensitivity (temp, light)	[164,165,166]
Chemiluminescence	Luminol, Lucigenin, L-012	O_2_^•−^, H_2_O_2_, peroxidase-driven reactions	Rapid detection of ROS in cells, plasma, tissues	Redox cycling (lucigenin), nonspecific interactions, antioxidant interference, possible false positives	[164]
Genetically encoded redox ligands	roGFP, HyPer, Grx1-roGFP2	H_2_O_2_, redox potential (GSH/GSSG)	Real-time imaging in cells; transgenic preclinical models	Requires genetic modification; potential disturbance of redox balance; limited clinical applicability	[167,168]
Nanoparticle-based ligands	Quantum dots, gold nanoparticles, ROS-responsive polymer NPs	ROS depending on surface chemistry: H_2_O_2_, O_2_^•−^, ^•^OH	In vivo imaging, targeted drug delivery, tumor redox profiling	Toxicity concerns, complex synthesis, biodistribution variability	[169]
Protein-based electrochemical ligands	Cytochrome-c, peroxidases, redox-active proteins	O_2_^•−^	Electrochemical biosensing platforms for real-time ROS detection	Requires robust immobilization; protein instability; selective detection depends on protein type	[164]
Biomarker-reactive ligands	DNPH (protein carbonyls), derivatization	Oxidized proteins, lipids, and DNA bases	Spectrophotometry, HPLC, LC-MS/MS, ELISA	Indirect detection; dependent on derivatization efficiency and sample handling	[162,170]

## Data Availability

No new data were created or analyzed in this study. Data sharing is not applicable to this article.
